# Broad‐scale acoustic monitoring of koala populations suggests metapopulation stability, but varying bellow rate, in the face of major disturbances and climate extremes

**DOI:** 10.1002/ece3.11351

**Published:** 2024-05-06

**Authors:** Bradley Law, Leroy Gonsalves, Traecey Brassil, Isobel Kerr

**Affiliations:** ^1^ Forest Science NSW Primary Industries Parramatta New South Wales Australia

**Keywords:** climate extremes, forestry, megafire, occupancy, passive acoustics

## Abstract

Population trends are lacking for most threatened species, especially those that are cryptic and difficult to survey. Recent developments in passive acoustics and semi‐automated call recognition provide a cost‐effective option to systematically monitor populations of vocal species. We assessed recent trends for the koala *Phascolarctos cinereus*, an iconic marsupial, based on 7 years of acoustic monitoring across 224 forested sites. The study period overlapped with a severe drought and extensive megafires in 2019 followed by 2 years of extremely high rainfall. Dynamic occupancy modelling with a range of covariates at multiple landscape scales found that initial occupancy was related to elevation (−ve), NDVI (+ve) and previous selective harvesting (16–30‐year age class; weakly +ve). Extinction probability increased with the extent of high‐severity fire. Colonisation probability was related to a range of factors, with the top model showing a decrease with increasing lagged annual rainfall. However, the null model was also supported, suggesting weak associations for colonisation. Using these relationships, koala occupancy was found to be high and stable over the study period. Fire did not influence regional trends because koalas often persisted with low‐ to moderate‐severity fire and because high‐severity fire was limited to 11% of their habitat. In contrast, bellow rate varied across years, being initially low and declining immediately after the 2019 fires, with the driver of these changes unclear. Neither timber harvesting nor low‐severity fire influenced koala occupancy or bellow rate. Given the extensive area of koala habitat in the region, our results point to the presence of a large population in these public forests, and in recent years, stable occupancy (albeit with site‐scale reductions in density with high‐severity fire). Ongoing monitoring is crucial for tracking future changes, especially with predictions of more frequent, severe forest fires due to climate change.

## Graphical abstract

Using acoustic monitoring and semi‐automated call recognition, we found the koala *Phascolarctos cinereus*, an iconic marsupial, maintained a stable trend in regional occupancy over 7 years. This was despite the study period overlapping with a severe drought and extensive megafires followed by 2 years of extremely high rainfall. Extinction probability increased with the extent of high‐severity fire, but neither low‐severity fire nor timber harvesting influenced trends in koala occupancy or bellow rate. Ongoing monitoring is needed for tracking future changes, especially with predictions of more frequent, forest fires from climate change.

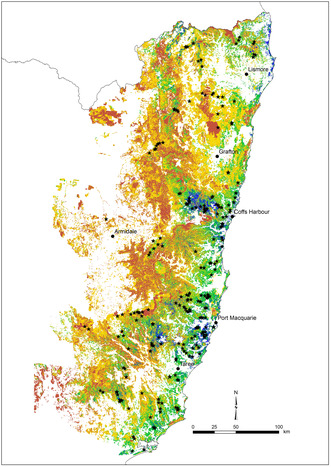

## 1 INTRODUCTION

Global climate change and the increasing incidence of large‐scale disturbances such as megafires have led to a heightened need for monitoring that can assess impact and recovery of key forest species (Jones et al., 2021; Legge et al., 2022). For example, the summer of 2019–2020 saw wildfires in eastern Australian forests that are the largest known in the historical record (Collins et al., 2021). The Australian Black Summer megafires comprised some 200 major fires that burnt through 10.4 million hectares (Mha) of land, including large areas of rainforest (0.33 Mha) (Godfree et al., 2021). Understanding the impacts of these fires and trajectory of recovery is critical for conservation planning as proliferation of these extreme climatic events is predicted to have severe consequences for animal populations, though empirical data tracking trends from pre‐ to post‐fire time periods are typically lacking (Legge et al., 2022).

Estimating population trends over adequate time periods are best placed to detect key drivers of demographic change and inform on the conservation status of threatened species (Marsh & Trenham, 2008). Yet the evidence‐base provided by long‐term trends is lacking for most species, especially those that are cryptic and difficult to survey. This hinders the implementation of effective conservation strategies if the key drivers of change are not targeted and understood (Caughley & Sinclair, 1994). Dynamic occupancy models (MacKenzie et al., 2003) have proved to be a powerful method for modelling change in occupancy probabilities over time, underpinned by potential drivers of colonisation and extinction and accounting for imperfect detection (Goldingay et al., 2022; Jones et al., 2021; Rodhouse et al., 2019). Moreover, occupancy is a useful surrogate for population assessment when density is low as is often the case for threatened species (MacKenzie & Nichols, 2004; McHugh et al., 2019).

The koala *Phascolarctos cinereus* is an iconic arboreal marsupial that occurs in the forests of eastern Australia. The Australian Government recently upgraded the conservation status of the koala from *vulnerable* to *endangered* (Department of Agriculture, Water and the Environment [DAWE], 2022), because of concerns about decline in various populations (McAlpine et al., 2015) and the impact of the recent megafires. Key threats to the koala are: permanent tree cover loss and fragmentation, increased housing around bushland, road traffic, dog attack, climate change (including megafires) and disease (McAlpine et al., 2015). Much of the basis for attributing decline derives from a combination of expert opinion and analysis of the historical record as well as direct habitat loss in many areas (Adams‐Hosking et al., 2016; McAlpine et al., 2015). Some repeat field surveys have demonstrated substantial declines in koalas in several areas, such as in western parts of their range due to climate extremes (Lunney et al., 2017) or extensive wildfire (Lunney et al., 2020), while citizen science snap‐shot surveys have also contributed to evidence of decline in some (Predavec et al., 2018), but not all (Lunney et al., 2016) areas.

Despite these contributions, annual systematic population trend data are mostly absent for koalas, and thus, any patterns of change over time are not easily discerned. Of two examples that assessed regular survey data, one using spotlighting and acoustics found a stable trend in occupancy that was unaffected by drought over 8 years (Goldingay et al., 2022) and another using diurnal transect counts found a decline in density between 1996 and 2014 in a region exposed to substantial urban development (Rhodes et al., 2015). Clearly, collecting annual data to document trends is an expensive process, especially for cryptic and often low‐density species such as koalas. However, new survey methods, especially passive acoustics, show great promise for cost‐effective monitoring of vocal species when combined with convolutional neural networks for automated detection of calls from recordings (Law et al., 2018; Ruff et al., 2021). Indeed because of their sensitivity and long deployment durations, passive acoustic monitoring can achieve high koala detection probabilities compared to alternative methods (Law et al., 2018). These attributes yield sufficient power to detect small changes in populations at landscape scales (Wood et al., 2019).

Our study aims were to analyse trends in regional koala occupancy across 0.83 million ha of public hinterland forests in north‐east New South Wales (NSW) using a dynamic occupancy framework. Acoustic monitoring at forest sites over a 7‐year period between 2015 and 2021 formed the basis of our dataset that spanned multi‐use state forests and conservation reserves. Male koalas bellow to advertise themselves in the spring breeding season (Ellis et al., 2011), so our monitoring focused on that season. Although public forests are exposed to fewer threats to koalas than private land (Gardiner et al., 2023; Law, Kerr, et al., 2022), extensive megafires burnt a substantial portion of the study area in 2019 following a severe drought. We assessed the impact of this large‐scale disturbance on the regional metapopulation and recovery in occupancy after fire, including the effects of fire severity. Previous occupancy analyses found only a slight negative effect for extent of wildfire, but severity was not considered in that study and few of the study sites had been burnt in the previous 10 years (Law et al., 2018). Also, acoustic arrays used to estimate koala density before and after fire found high and moderate severity fire had a large impact on density, while low‐severity fire had no detectable effect (Law, Gonsalves, Burgar, Brassil, Kerr, & O'Loughlin, 2022). Based on these results we predicted high‐severity wildfire would lead to an increased probability of local extinction. Regulated timber harvesting occurs in state forests, but unlike megafires, it is dispersed in space and time, and previous occupancy analyses have found no relationship with time since harvest or harvest intensity (Law et al., 2018). Moreover, a before‐after‐control‐impact study found no evidence for an impact of harvesting on koala density (Law, Gonsalves, Burgar, Brassil, Kerr, O'Loughlin, et al., 2022) and radio‐tracking confirms koalas commonly use areas regenerating soon after timber harvesting (Law, Slade, et al., 2022). Thus, we predict that this disturbance is unlikely to influence trends in occupancy at local or regional scales. We also assessed bellow rate, a potential index of koala abundance (Hagens et al., 2018), and its relationship with environmental covariates because it is a potentially more sensitive metric than occupancy. Bellow rate is an index of activity that is correlated with density in at least some populations (Hagens et al., 2018), although it may also vary behaviourally.

## 2 MATERIALS AND METHODS

### 2.1 Study area

**FIGURE 1:**
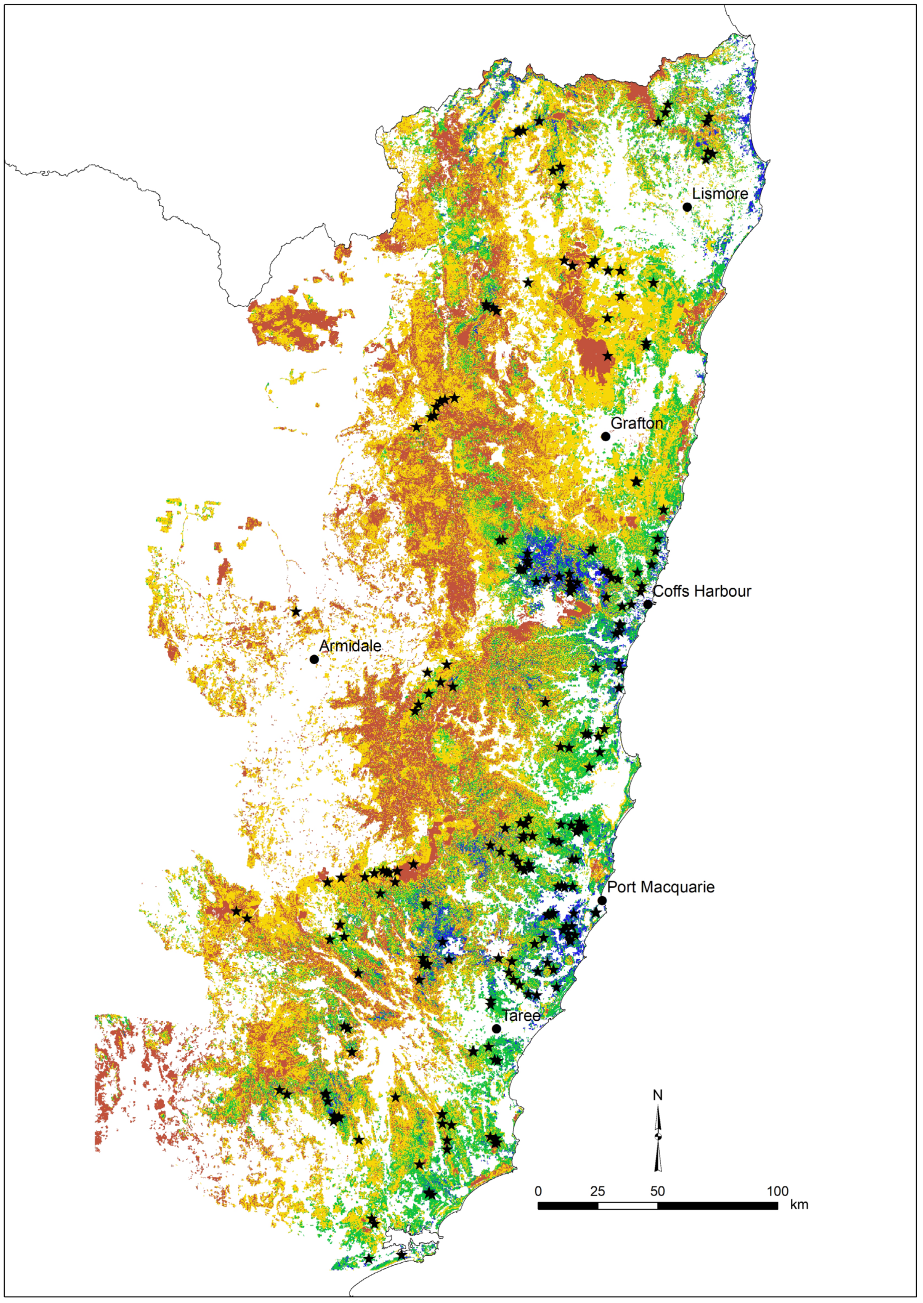
Map showing 224 monitoring sites overlayed on koala habitat suitability. Blue and green show higher suitability; yellow and red show lower suitability.

The study area spanned native forests of the hinterland, ranges and tablelands of north‐east NSW bounded by the Hunter River in the south, the Queensland border in the north and Armidale in the west (~8.5 Mha) (Figure 1). The coastal strip was generally excluded as koalas in this area are exposed to multiple threats associated with urbanisation, which does not affect forests in our study. Both State forests and National Parks were sampled, while private land (including private forestry) was excluded (but see Law, Kerr, et al., 2022). The study region supports a rich diversity and mosaic of forest types, from tall moist eucalypt forest to dry sclerophyll forests and woodlands, including a range of important koala browse tree species (Moore et al., 2004; Phillips et al., 2000).

### 2.2 Monitoring design and black summer fires

Koalas were monitored annually using passive acoustics between 2015 and 2021 at a total of 224 sites. Sites encompassed a broad range of forests stratified by koala habitat suitability based on MaxEnt models for the study region (Law, Caccamo, et al., 2017), but those receiving the most repeated visits targeted modelled medium to high suitability. In the first 3 years of the study (2015–2017), sites were rotated so that they were surveyed once each as they were established for a snapshot occupancy survey for koalas in the study area (Law et al., 2018). Therefore, we pooled data from these years to provide a robust estimate of initial occupancy across the 224 sites, because establishing an appropriate baseline is important given occupancy estimates for subsequent years are derived (using local colonisation and extinction) from the initial baseline (2015–2017). The low metabolic lifestyle of koalas (Ellis et al., 2009) should result in little change in populations over this short time frame. Thereafter, a subset of the initial sites (50–60) was revisited for monitoring each year with these varying depending on access and influence of wildfire (see below).

A power analysis confirmed that an annual −3.89% (−30% in 10 years) decline in koala occupancy can be detected with 80% power by monitoring 61 sites per year (seven nights of survey) if koala habitat is targeted and, for dynamic occupancy, that there was little benefit in having >30% of all sites sampled each year as annual sites (Kavanagh et al., 2022).

The initial survey (2015–2017) aimed to sample a broad range of timber harvest intensities and times since harvest as well as mapped old growth (Law et al., 2018). We intentionally sampled a range of topographic locations (gullies to ridge tops), forest types and elevations (10–1327 m above sea level [ASL]). Site locations were initially selected using GIS to sample a range of forest harvesting treatments in each sampling session, though this was sometimes modified in the field if GIS mapping proved inaccurate.

In 2019, an intense drought in south‐eastern Australia precipitated extensive spring‐summer megafires that burnt an absolute area of high‐severity fire (~1.8 Mha) that was larger than ever previously recorded in one fire season (Collins et al., 2021). Of the 1.65 Mha of better‐quality koala habitat in north‐east NSW (Law, Caccamo, et al., 2017), 70% remained unburnt, 7% experienced low‐severity fire, 12% had moderate severity burn and 11% high fire severity burn. Given about 30% of koala habitat was burnt across the region, we ensured that our monitoring sites were representative of the fire extent for koalas by sampling a similar proportion (~30%) of burnt sites. We also note that the fires in northern NSW commenced in early spring 2019, 1–2 months prior to our sampling in that year.

### 2.3 Acoustic sampling

At each site, we deployed one Song Meter (SM2+ in 2015, SM4 in 2016–2019, SM mini in 2020–2021–Wildlife Acoustics, Maynard USA) to record koala bellows. AudioMoths (version 1.1; Hill et al., 2019) were used to sample four sites. Sensors were programmed to record from sunset until sunrise, the peak calling period of koalas (Ellis et al., 2011) (SM4: sampling rate 22 kHz, resolution 16 bits per sample, gain 16 db; SM‐mini: sampling rate 22 kHz, gain 18 dB). We deployed Song Meters for at least seven consecutive nights (7–24 nights) in the breeding season (mid‐August to December) in each year between 2015 and 2021. Previous analyses showed that this level of survey effort (at least seven nights) has little impact on detection probability because of the high detection rate per night.

Call playback in forests at 75 dB has found that call amplitude in recordings attenuates to background noise levels within 100–150 m (Charlton et al., 2012; Hagens et al., 2018), although we expect under ideal conditions this could extend to ~300 m (Law et al., 2021). Temperature could also affect bellowing frequency or sound propagation (Law et al., 2018), and we account for this by modelling month (related to temperature) as a covariate for detection probability (see below). We also note that recording devices detect sounds over shorter distances than human ears and detection algorithms are less efficient for faint calls (Yip et al., 2017).

Research was undertaken with a NSW Department of Planning and Environment Scientific Licence (SL 100623).

### 2.4 Automated analysis of koala bellows

Recordings were scanned using koala recognisers. Those collected pre‐fire in 2015–2018 were scanned with Ecoacoustics Audio Analysis (version 1 or 2; Towsey et al., 2012), while recordings collected post‐fire in 2019–2021 were scanned by AviaNZ acoustic software (Marsland et al., 2019) using a koala recogniser (https://www.dpi.nsw.gov.au/forestry/science/forest‐ecology/fauna‐identification‐service/metadata‐information‐for‐koala). We made this change because the validation process was faster in AviaNZ. Although recognisers varied in recall and precision, we assessed the effect of this by including recogniser version as a covariate for detection probability (see below). Recordings matched by the koala recogniser were validated by manually visualising spectrograms of the audio and listening to recordings to check for false positives. A single koala bellow often comprised multiple event triggers. The mean length of a koala bellow has been reported as 36 s and few last more than a minute (Ellis et al., 2011; Smith, 1980).

A detection history matrix was developed for each site such that a night with >0 koala bellows was assigned a detection (‘1’) and a site with no detected bellows on a night was assigned a non‐detection (‘0’). To quantify the bellowing activity, a putative index of koala abundance, and allow for some independence between bellows, we summed the number of 10‐min periods per night with a koala bellow.

### 2.5 Habitat and GIS covariates

A geographic information system (ArcMap 10.7.1, ESRI) was used to classify the surrounding landscape of each site within a 500 m and 1 km buffer, noting 1 km landscapes had the greatest previous support for koala occupancy (Rhodes et al., 2008; Table 1). These GIS covariates included the extent and severity of wildfire in the last 2 years (to address immediate impact) based on the Fire Extent and Severity Mapping (FESM; Gibson et al., 2020) with severity ratings pooled into three categories, being unburnt (category 0), low fire‐severity (categories 2 and 3) and high fire‐severity (categories 4 and 5); area harvested for timber <5 years ago, 5–15 years, 16–30 years and >30 years and the extent of old growth (Table 1). Low‐intensity hazard reduction fires were not included in analyses because they affected <3.5% (and often 0%) of 500 m site buffers in any given year. We also derived site productivity by calculating normalised difference vegetation index (NDVI – normalised difference vegetation index, Pettorelli et al., 2011) values using MODIS MOD13Q1 granules averaged for July–September in each year sampled with this time period coinciding with the driest time of year and thus productivity immediately preceding our survey season. We sampled two levels of harvest intensity: light‐moderate selective (<80 m^3^ timber removed per compartment [~250 ha]) and heavy harvesting (>80 m^3^ timber removed). Harvest intensity was designated based on recent Geographic Information System (GIS, ArcMap 10.4.1, ESRI) layers (GISO.EventPoly_State; FCNSW, unpubl. data) or, for older harvesting (prior to 2001), using a Management History layer containing volumes of timber removed from compartments (FCNSW, unpubl. data). Extent of harvest intensity for different times since harvest was calculated for each buffer size. Annual rainfall and the annual mean of the daily maximum temperature, each lagged by 1 year prior to sampling, were extracted for a 500 m buffer around sites using SILO interpolation of weather data (https://www.longpaddock.qld.gov.au/silo/; Table 1). Long‐term climate data experienced at each site was derived from ANUCLIM for mean annual precipitation and mean annual temperature (Table 1).

**TABLE 1:** Description of covariates used to model variation in *ρ* (detectability), Ψ1 (initial occupancy), *γ* (colonisation) and *ε* (extinction).

Variable	Description	*ρ*	Ψ1	*γ*	*ε*
Sensor type	SM2, SM4, SM‐mini, AudioMoth	✓			
Recogniser version	Ecosounds version 1, Ecosounds version 2, AviaNZ (CNN15)	✓			
Month	Month of survey	✓			
Season	Year of survey	✓			
Climate					
Lagged annual rainfall	Total annual rainfall over previous 12 months (SILO interpolation), 500 m resolution	✓	✓	✓	✓
Lagged annual maximum temperature	Annual mean of the daily maximum temperature over previous 12 months (SILO interpolation), 500 m resolution	✓	✓	✓	✓
Precipitation (long‐term)	ANUCLIM, mean annual precipitation in 1 km & 500 m buffer		✓		
Mean annual temperature (long‐term)	ANUCLIM, mean annual temperature in 1 km & 500 m buffer		✓		
Environmental					
DEM	Site elevation (m ASL), mean of 1 km & 500 m buffer		✓		
Habitat model	Mean of DPI koala model in 1 km & 500 m buffer		✓		
Land tenure	State forest versus national park/reserve		✓		
Soil fertility	Mean % of total phosphorous at 0 to 5 cm in 1 km & 500 m buffer		✓		
Roughness (100)	Neighbourhood Topographical Roughness based on Standard Deviation of Elevation in circular 100 – m Neighbourhood Mean of roughness in 1 km buffer & 500 m buffer, 30 m resolution		✓		
Roughness (1000)	Neighbourhood Topographical Roughness based on Standard Deviation of Elevation in circular 1000 – m Neighbourhood Mean of roughness in 1 km & 500 m buffer, 30 m resolution		✓		
Topographic Position	Mean Topographic Index in 1 km buffer & 500 m buffer		✓		
Landscape old growth	% area of mapped old growth in 1 km & 500 m buffer		✓		
Landscape rainforest	% area of mapped rainforest in 1 km & 500 m buffer		✓		
SMIPS	Soil Moisture Information Processing System (CSIRO); Index of moisture in the top 90 cm & 1 km resolution over previous 12 months		✓	✓	✓
NDVI	NDVI value (mean July–September in survey year) in 1 km & 500 m buffer		✓	✓	✓
Disturbance					
Landscape heavy harvesting (<5 years)	% area of heavy harvesting (<5 years) in 1 km & 500 m buffer		✓	✓	✓
Landscape selective harvesting (<5 years)	% area of selective harvesting (<5 years) in 1 km & 500 m buffer		✓	✓	✓
Landscape heavy harvesting (5–15 years)	% area of heavy harvesting (5–15 years) in 1 km & 500 m buffer		✓	✓	✓
Landscape selective harvesting (5–15 years)	% area of selective harvesting (5–15 years) in 1 km & 500 m buffer		✓	✓	✓
Landscape heavy harvesting (16–30 years)	% area of heavy harvesting (16–30 years) in 1 km & 500 m buffer		✓	✓	✓
Landscape selective harvesting (16–30 years)	% area of selective harvesting (16–30 years) in 1 km & 500 m buffer		✓	✓	✓
Landscape heavy harvesting (>30 years)	% area of heavy harvesting (>30 years) in 1 km & 500 m buffer		✓	✓	✓
Landscape selective harvesting (>30 years)	% area of selective harvesting (>30 years) in 1 km & 500 m buffer		✓	✓	✓
Landscape wildfire (high severity)	% area of high‐severity wildfire 2019/2020 Black Summer Fires in 1 km & 500 m buffer		✓	✓	✓
Landscape wildfire (low severity)	% area of low‐severity wildfire 2019/2020 Black Summer Fires in 1 km & 500 m buffer		✓	✓	✓

### 2.6 Occupancy modelling

A dynamic occupancy modelling framework (MacKenzie et al., 2003) was used to estimate changes in koala occupancy between 2015–2017 and 2021. Detection data used in the modelling were derived from 224 sites sampled, with not all sites sampled in each year.

A hierarchical approach was taken to modelling to reduce the total number of candidate models. Detection probability (*ρ*) was first modelled to account for imperfect detection associated with surveys, and initial occupancy, colonisation and extinction were held constant. Covariates for modelling *ρ* included sensor type, recogniser version, month, season (i.e. year of sampling), annual rainfall and mean maximum temperature (Table 1). Previous analyses suggested that detection of the low‐frequency bellow of koalas is not strongly influenced by variation in topographic locations or harvest history of Song Meters (Law et al., 2018; B. Law, G. Gonsalves and T. Brassil, unpubl. data). Although background noise levels (e.g. during rain) may reduce detection, our previous analyses have found it either had no effect (Law, Kerr, et al., 2022) or only a minor effect on koala detection probability (Law et al., 2018), probably because we aimed to avoid heavy rain and seven nights of sampling typically yields >90% detection probability. Accordingly, we omitted survey rainfall from our analyses. A null model that assumed constant detection across all visits to a given site was also included in the candidate set of models. The top model was carried forward to model initial occupancy (i.e. occupancy in 2015–2021).

Initial occupancy (Ψ1) was modelled while holding colonisation and extinction constant. Several covariates were modelled based on a range of site‐based variables that were either previously shown to be related to koala occupancy or are hypothesised to be influential (Gardiner et al., 2023; Law et al., 2018; Law, Kerr, et al., 2022) or (Table 1). These included elevation (m ASL), mean annual temperature, mean annual rainfall, NDVI, modelled habitat suitability, soil phosphorus, soil moisture, topographic roughness, topographic position (potentially a surrogate for distance to water), tenure and the extent of wildfire (in previous 2 years), extent of timber harvesting (<5 years, 5–15 years, 16–30 years and >30 years), extent of mapped high conservation value old growth, extent of rainforest and land tenure (state forest or national park estate). A null model that held initial occupancy constant across sites was also included in the set of candidate models. The influence (direction and magnitude) of a supported covariate was assessed by plotting occupancy estimates that were generated while holding all other supported covariates at the median value (for continuous variables) or mode (for categorical variables).

Colonisation (*γ*) and extinction (*ε*) parameters were then modelled separately using the top model for initial occupancy and while holding the other dynamic parameter constant. Variables included as covariates for these parameters were the extent and severity of wildfire in the previous 2 years (FESM), the extent (%) of timber harvesting (<5 years, 5–15 years, 16–30 years and >30 years ago), total rainfall for the 12 months preceding each annual survey (from Bureau of Meteorology SILO Climate Database) and NDVI (September of each year). A null model where these parameters were held constant was also included.

The trend for koala occupancy was estimated by first calculating initial occupancy in 2015–2017 assuming median conditions for supported covariates among all sites. Occupancy estimates for subsequent years were derived based on relationships established between covariates and colonisation and extinction. For each subsequent year, the median value for supported covariates in that year was used to establish the proportion of occupied sites that became locally extinct and the proportion of unoccupied sites that were colonised. This provided a trend for the study area (i.e. across all sites) rather than for sites sampled in each year which may be biased if annual, but partial, sampling is not representative of the range of conditions in the study area.

Prior to analysis, covariates were standardised and examined for collinearity. All covariates were initially included in the models, but if any highly correlated (*r* > .7) variables appeared in the same model only one covariate was retained and the model was re‐run. This was most commonly the case when 500 m and 1 km buffers for a covariate were used in modelling as these were often correlated. Only the most supported buffer for a covariate was retained in the final model. The only other correlated variables were DEM (elevation) and mean annual long‐term temperature and these two variables were not included in the same model. Modelling and model selection were carried out in program R using the RPresence package (MacKenzie & Hines, 2021).

When modelling occupancy, a staged approach was taken whereby single covariates were first assessed. The top single covariate model (*n*) was then built upon by including an additional covariate in a 2‐covariate additive model. If a 2‐covariate model (*n* + 1) improved on the AIC score of model *n* by >2 AIC points, it was retained and carried forward in further modelling that included additional covariates (i.e. a 3‐covariate model and so on). This process was continued until the addition of an extra covariate did not improve the AIC score by >2 AIC points.

Lastly, supported candidate models for detection probability (*p*) and initial occupancy (Ψ1) were model‐averaged to provide estimates of all parameters. Supported models were those that were within 2 AIC points of the top model, for both detection and occupancy. Only the top models were plotted for extinction and colonisation because model‐averaging was not possible for these parameters in dynamic occupancy models using the RPresence package.

We assessed the fit of the models to the data by using the method of MacKenzie and Bailey (2004) for each year separately since a goodness of fit test was not available for dynamic occupancy models in RPresence. The most general single‐season occupancy model and 10,000 bootstrap samples were used when assessing fit. The test statistic indicated that there was no lack of fit to the data for all years (*p* > .05).

### 2.7 Modelling koala bellow rate (N‐mixture modelling)

We used stacked N‐mixture models to assess associations between koala bellow rate (number of 10 min periods with a bellow night^−1^ site^−1^ to improve data independence following the approach for camera traps by Scoleri et al., 2023) and covariates. Using such an approach, data collected in subsequent years are stacked so that unique site‐year combinations are treated as separate sites. To reduce potential impacts of pseudoreplication, site was included as a random effect in all models for bellow rate. Sites at which a koala was not detected after multiple years of sampling were considered to be absent given the high probability of detection for recording at least one bellow after seven nights of sampling. These sites were generally of low habitat quality and were excluded from modelling because absent sites are uninformative for understanding variation in bellowing activity. Sites included in the analysis were modelled as having moderate to high MaxEnt‐modelled suitability (Law, Caccamo, et al., 2017) that was on average >0.45 within 500 m. Like occupancy modelling, N‐mixture models detection probability and adjusts for imperfect detection of bellows when estimating bellow rate. Detection probability was assessed against year of survey, month of survey, sensor type and recogniser version. A null model which held detection constant was also assessed. The most supported model for detection probability was carried forward when modelling bellow rate. The same covariates used in occupancy modelling were assessed when modelling bellow rate (Table 1). Model selection for both detection probability and bellow rate used leave‐one‐out cross‐validation (LOO) (Vehtari et al., 2017) via the loo package (Vehtari et al., 2023). The model with the largest expected predictive accuracy (‘elpd’) performed best. The top model was retained if the difference (‘elpd_diff’) between the ‘elpd’ of the next model and the top model was >4. The top model was then carried forward and a 2‐covariate additive model was assessed using the remaining covariates. This process continued until the addition of a covariate did not improve on the top model. All covariates were standardised prior to analysis. Modelling was undertaken using the UBMS (Kellner et al., 2022) package in R.

## 3 RESULTS

Monitoring with Song Meters produced an extensive dataset that amassed >25,000 h of nocturnal recording over >2500 nights with >11,000 bellows identified. Koalas were detected on 312 site surveys (67%) in the hinterland forests ranging from 29% naïve occupancy in 2015 (using SM2 Song Meters) to 82% in 2018 and 2019 (mostly SM4 Song Meters).

### 3.1 Detection probability

**FIGURE 2:**
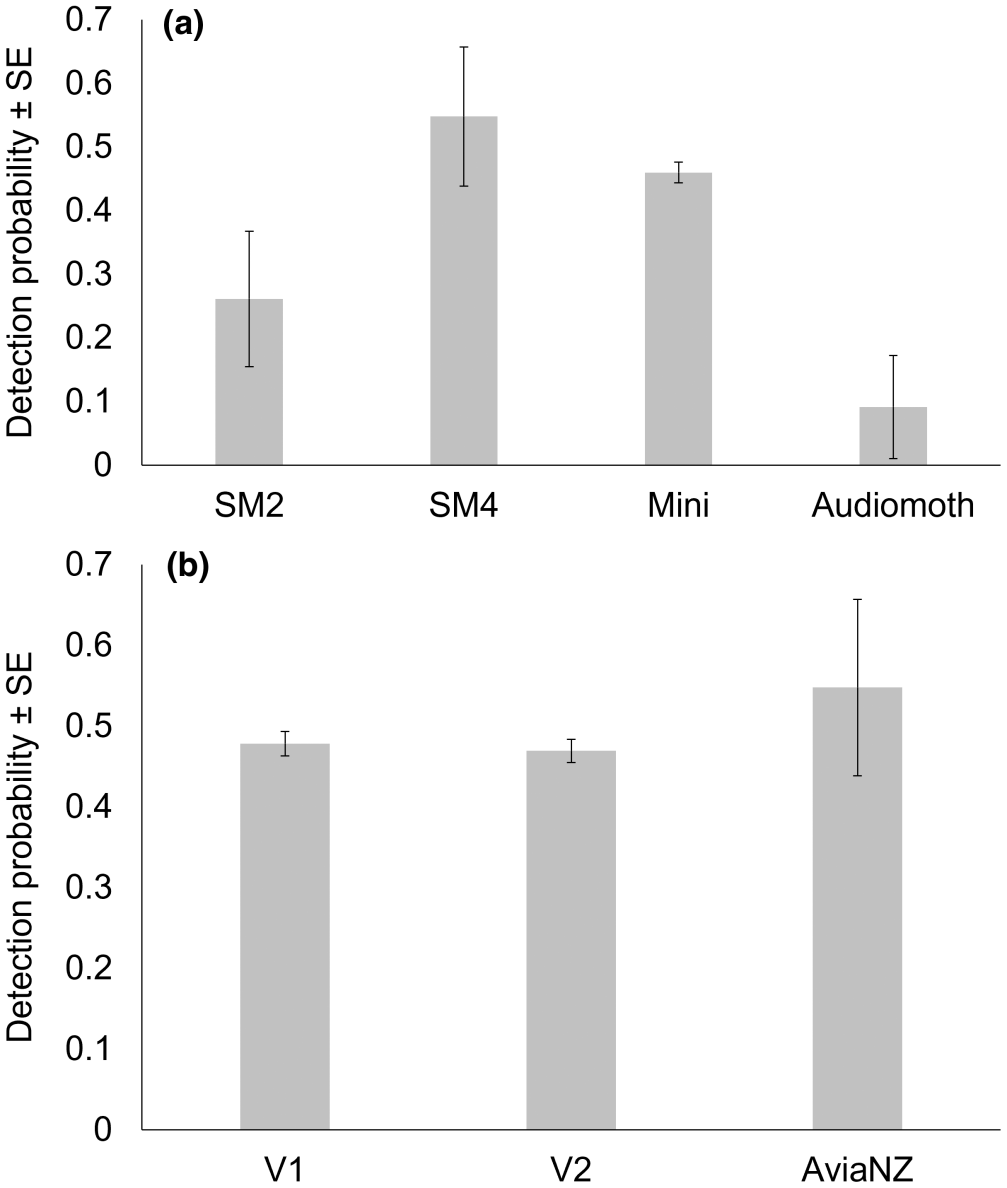
The influence of (a) sensor type using AviaNZ recogniser and (b) recogniser version on nightly detection probability using SM4. Error bars are 95% confidence limits.

In all, nine candidate models were fitted for detection probability (Table S1). Two models were supported. The top model allowed detection probability to vary with sensor type, whereas the second most supported model also included recogniser as an additive variable (Table S2). SM4 units had the highest detection probability per night and AudioMoths the lowest (Figure 2a), while the effect of recogniser version was relatively minor (Figure 2b). A detection probability of >90% was achieved by sampling for a minimum of seven nights for SM4s. All other covariates were not supported.

### 3.2 Initial occupancy (2015–2017)

**FIGURE 3:**
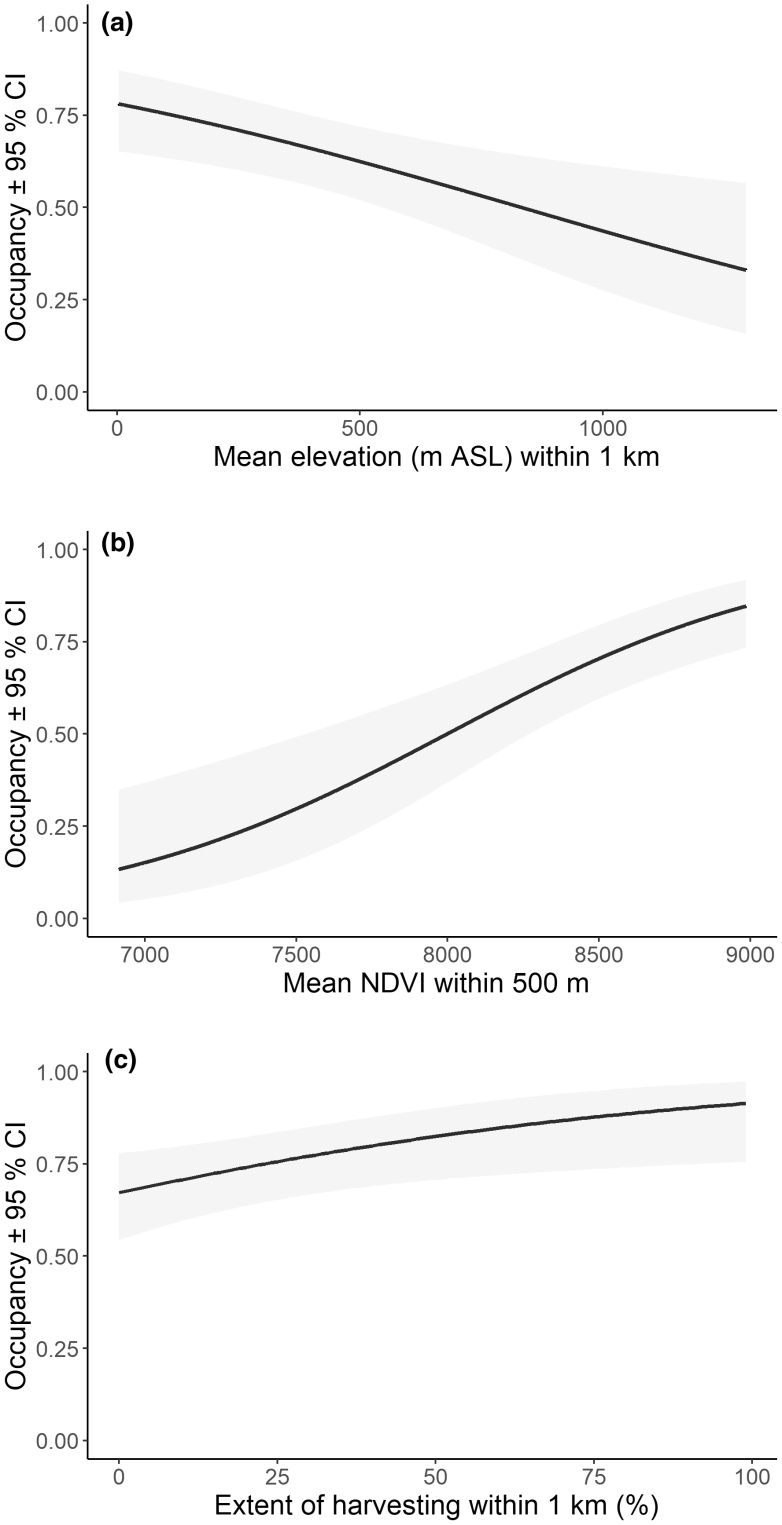
The influence of (a) elevation (m) (1 km buffer), (b) NDVI (500 m buffer) and (c) extent of selective harvesting (16–30 years previously) (1 km buffer) on initial occupancy. Shading shows 95% confidence limits.

In all, 97 candidate models were fitted to the data to assess initial occupancy by koalas (Table S2). A single model was supported. This model allowed initial occupancy to vary with elevation (mean elevation within 1 km), NDVI (mean NDVI within 500 m) and the extent of medium‐intensity harvesting 16–30 years previously (within 1 km). Elevation had the strongest effect on initial occupancy, with occupancy being very high at lower elevations (~0.8) and declining to <0.5 above 1000 m (Figure 3a). Occupancy also increased positively with NDVI (Figure 3b), and a weaker positive relationship was also supported for the extent of selective intensity harvesting 16–30 years previously (Figure 3c).

### 3.3 Colonisation

**FIGURE 4:**
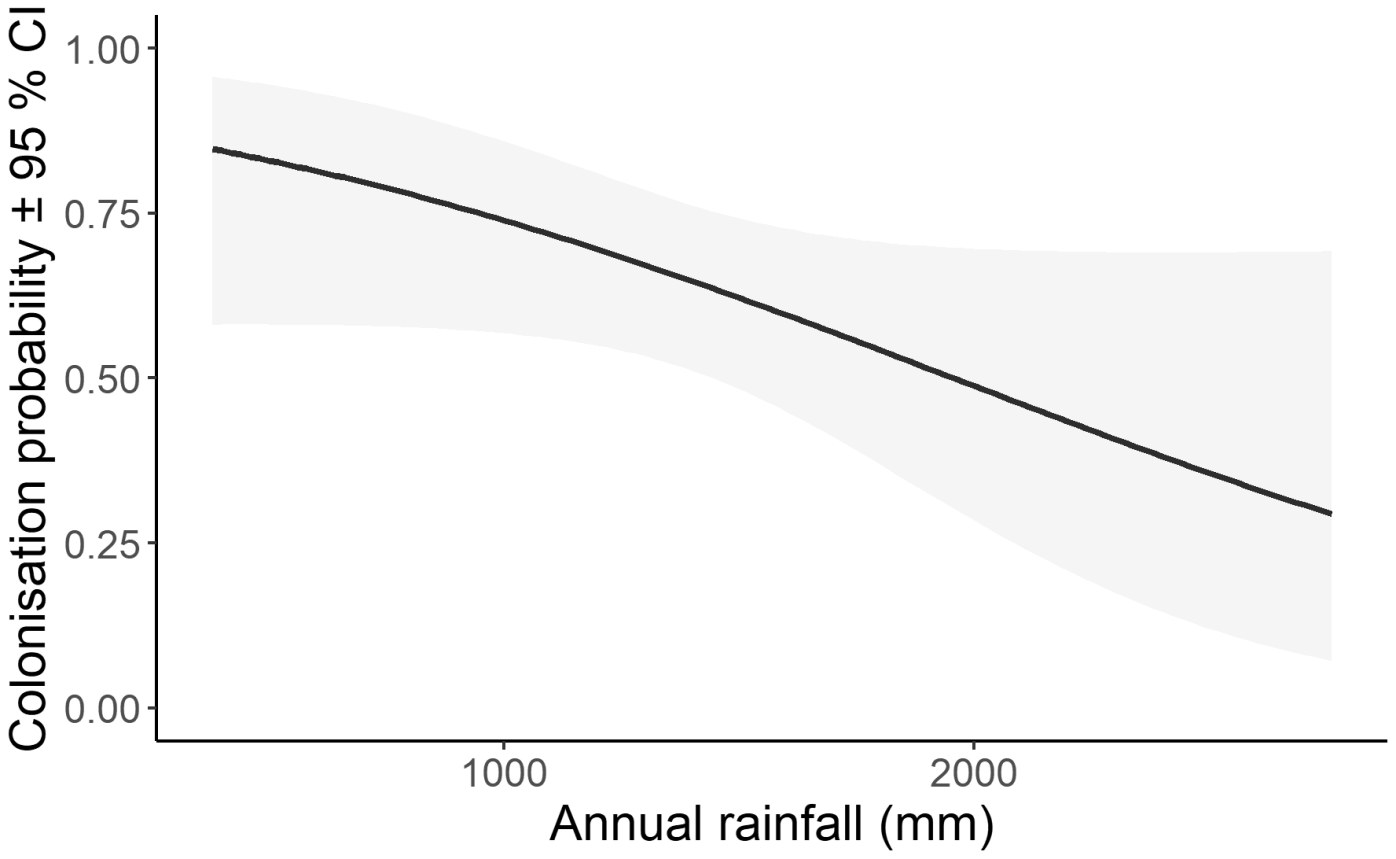
Relationship between colonisation probability and lagged annual rainfall. Shading shows 95% confidence limits.

In all, 27 candidate models assessed colonisation probability for the koala (Table S3). Of these, 14 models were supported, including the null, indicating weak associations between supported covariates and colonisation probability. The top model allowed colonisation probability to vary with annual rainfall preceding surveys at each site. Colonisation probability decreased from 0.65 at sites with the lowest annual rainfall (380 mm) to 0.29 at sites with the highest annual rainfall (2760 mm) (Figure 4). Under median conditions across the monitoring program, colonisation probability was 0.62 ± 0.07 per year.

### 3.4 Extinction

**FIGURE 5:**
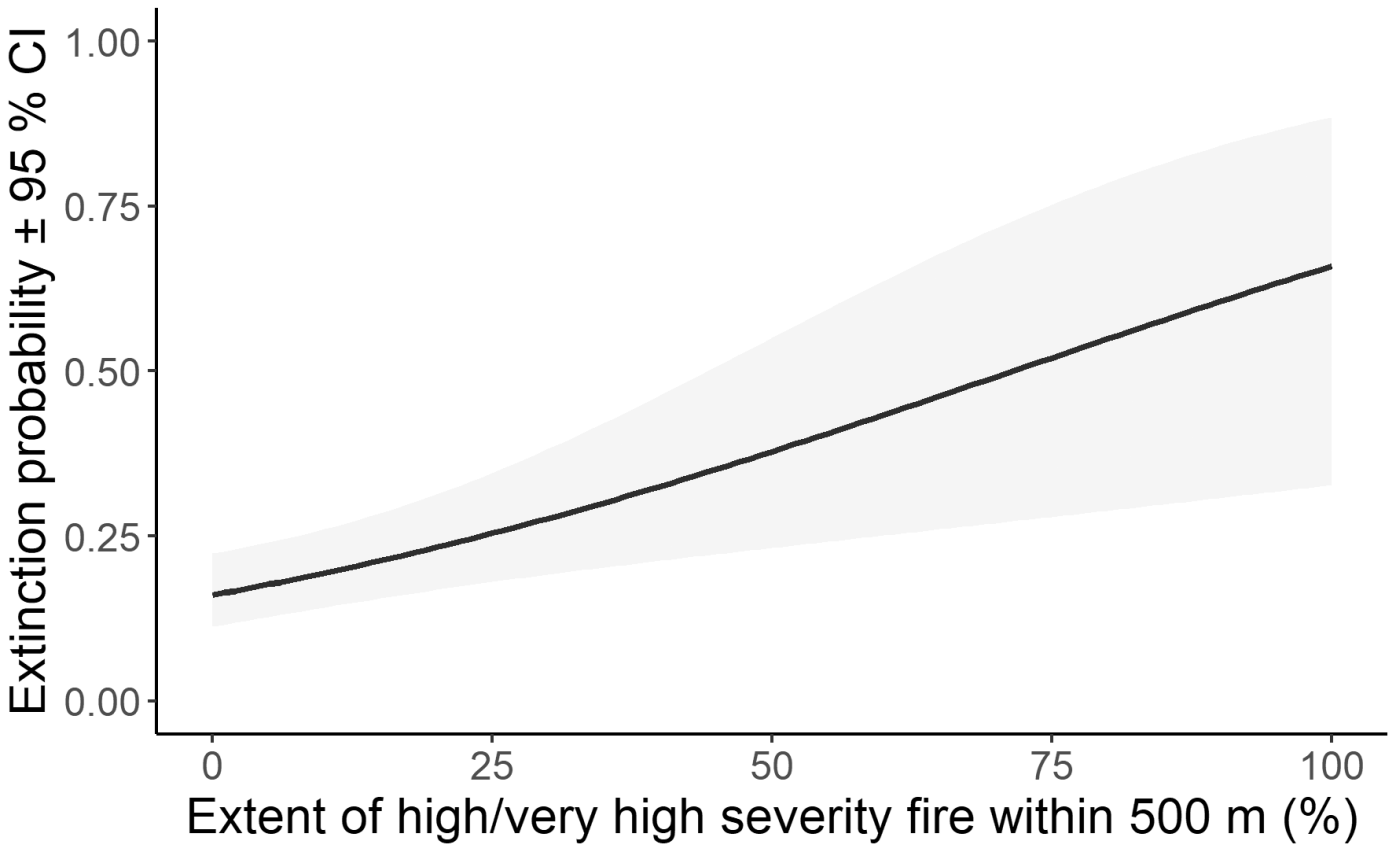
Relationship between extinction probability and the extent of high‐severity fire within a 500 m buffer of the site.

**FIGURE 6:**
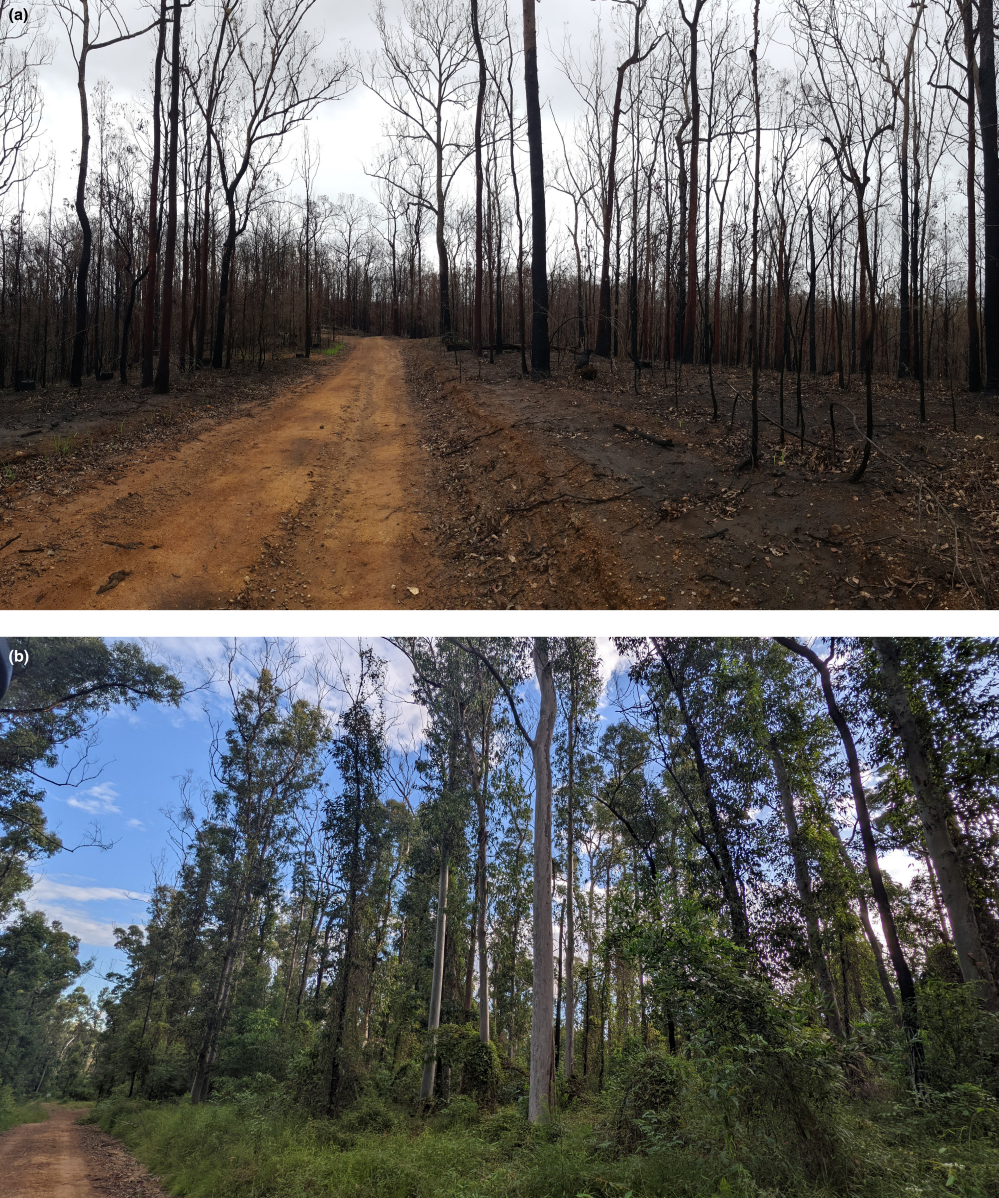
Portion of Kiwarrak State Forest burnt by high‐severity fire in 2019 (above, a) and canopy recovery in 2022 (below, b).

**FIGURE 7:**
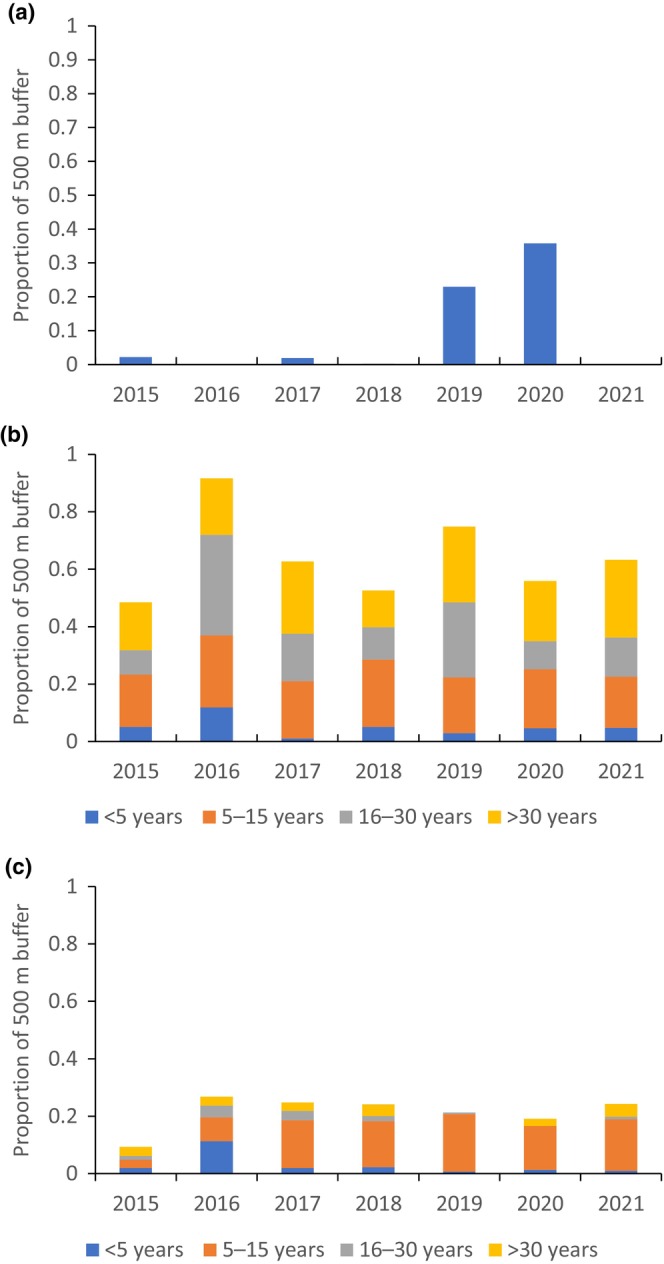
Proportion of site buffers (500 m) affected by (a) wildfire in the past 2 years (all sites), (b) selective harvesting in different age classes (state forest only) and (c) heavy harvesting in different age classes (state forest only). Note that harvesting exclusions have increased over time (meaning a greater proportion of the landscape was harvested in older age classes) and areas of selective harvesting can have repeat harvests in the same area, inflating the actual areal extent plotted.

The extent of high‐severity fire surrounding sites was the only covariate supported to influence extinction probability (Table S4). Extinction probability increased from 0.16 at sites unaffected by high‐severity fire to 0.66 where 100% of a site's buffer (500 m) was burnt by high‐severity fire in the previous 2 years. During the Black Summer fires in 2019, 17 sites were burnt with mixed severity in the months prior to surveys, which amounted to 36% of the 500 m site buffers (Figure 5). Ten sites mostly experienced high‐severity fire and seven mostly experienced low‐severity fire. Canopy recovery occurred within 2 years in many areas (Figure 6). Prior to 2019, few of our study sites had wildfires within the previous 2 years (Figure 7a).

Low‐severity fire extent, time since timber harvesting and its extent as well as lagged annual rainfall were not supported as covariates for extinction probability. Selective timber harvesting covered a greater extent than heavy harvesting at state forest sites (500 m buffer), with heavy harvesting in the 0–5 year category covering a maximum of 11% of the site buffers in any 1 year, reducing to <1% after the Black Summer fires of 2019 (Figure 7). Overall, 41% of sites (92 of 224) had some level of recent harvest between 2015 and 2021.

### 3.5 Trend in occupancy

**FIGURE 8:**
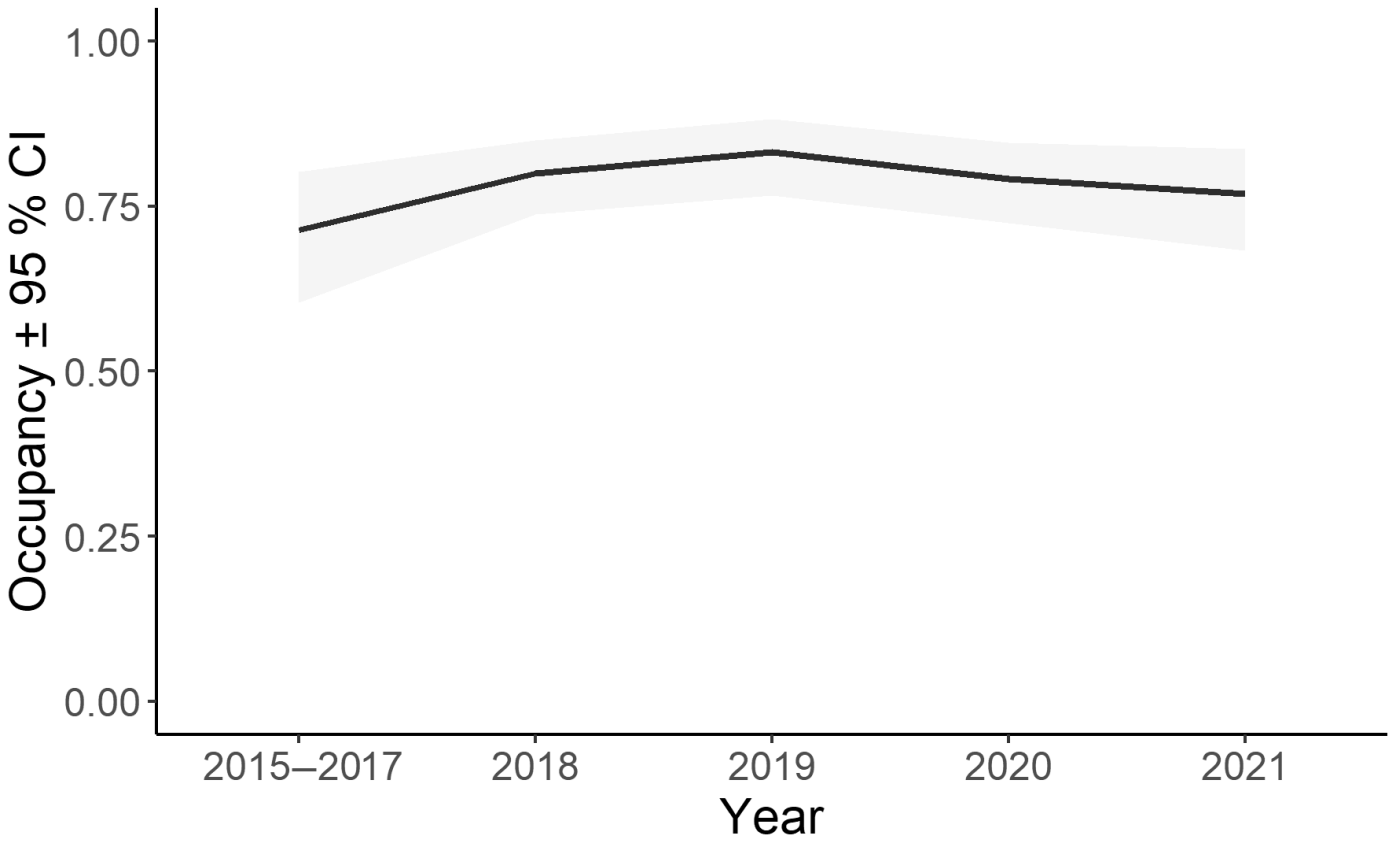
A stable trend in regional koala occupancy after accounting for imperfect detection and the environment at monitoring sites. Estimates for each year assume median values; i.e. an elevation of 231 m ASL, NDVI score of 0.8530 and selective harvesting (16–30 years age class) over 8% of a 1 km site buffer.

Koala occupancy remained high and stable between 2015–2017 and 2021 (Figure 8). Estimates for each year following 2015–2017 are made assuming median conditions for all supported covariates (i.e. elevation, NDVI, extent of medium‐intensity harvesting (16–30 years) and extent of high‐severity fire) and lagged annual rainfall. There was no appreciable decline in 2019 after the Black Summer fires despite high‐severity fire being associated with higher extinction probability, although a slight decline within confidence limits was evident during 2020 and 2021. Overall stability likely results from ~70% of hinterland koala habitat remaining unburnt or burnt by low‐severity fire. Our post‐fire monitoring sites represented a similar proportion of burnt habitat (~30%).

### 3.6 Bellow rate

**FIGURE 9:**
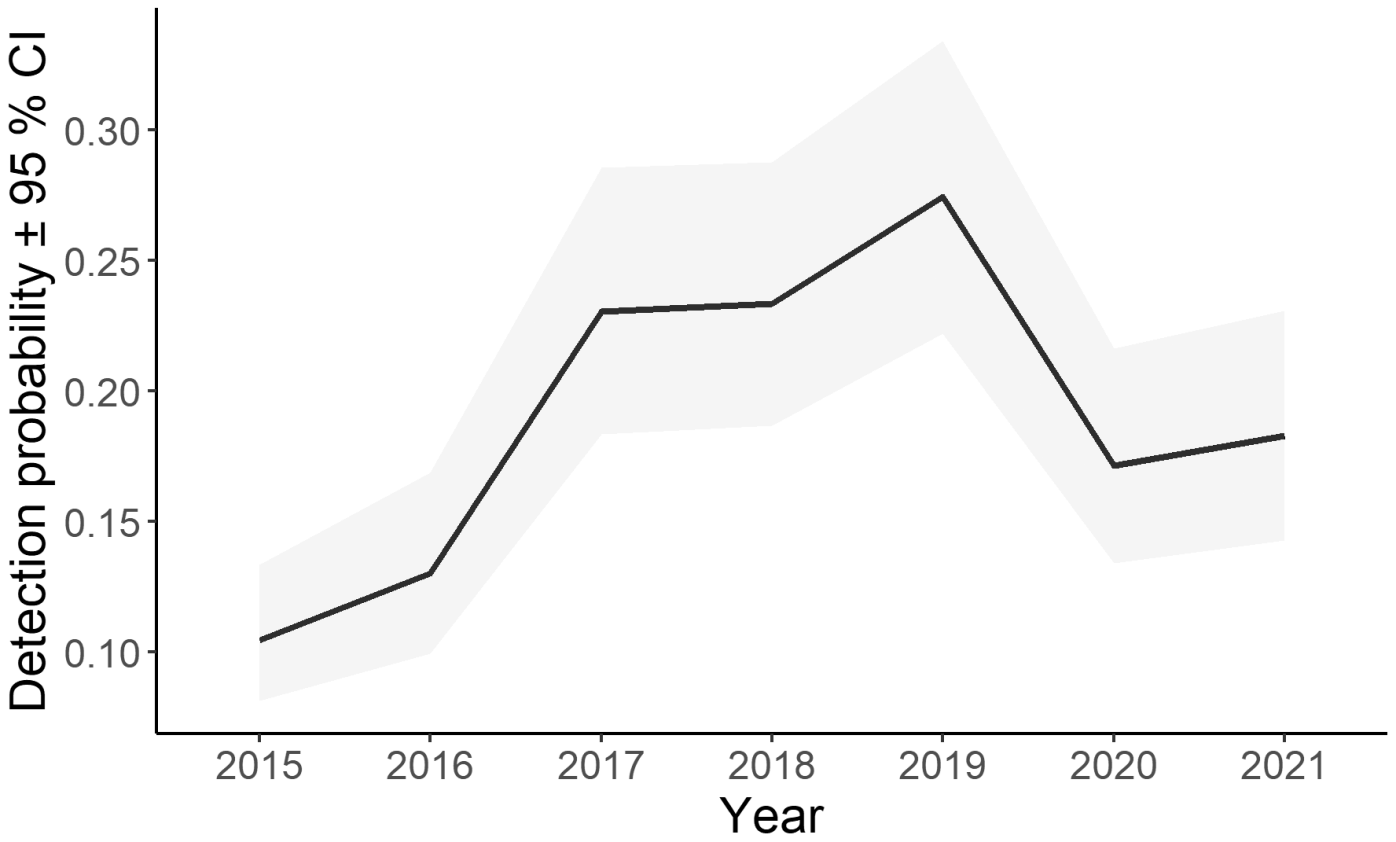
Line graph illustrating nightly detection probability for individual koala bellows among years.

In total, 10,610 10 min periods were recorded as having a koala bellow. Two models had support for detection probability of these (Table S5). The top model allowed detection to vary with year of sampling. Detection probability for individual bellows varied over the years and was lowest in 2015 and highest in 2019 (Figure 9).

**FIGURE 10:**
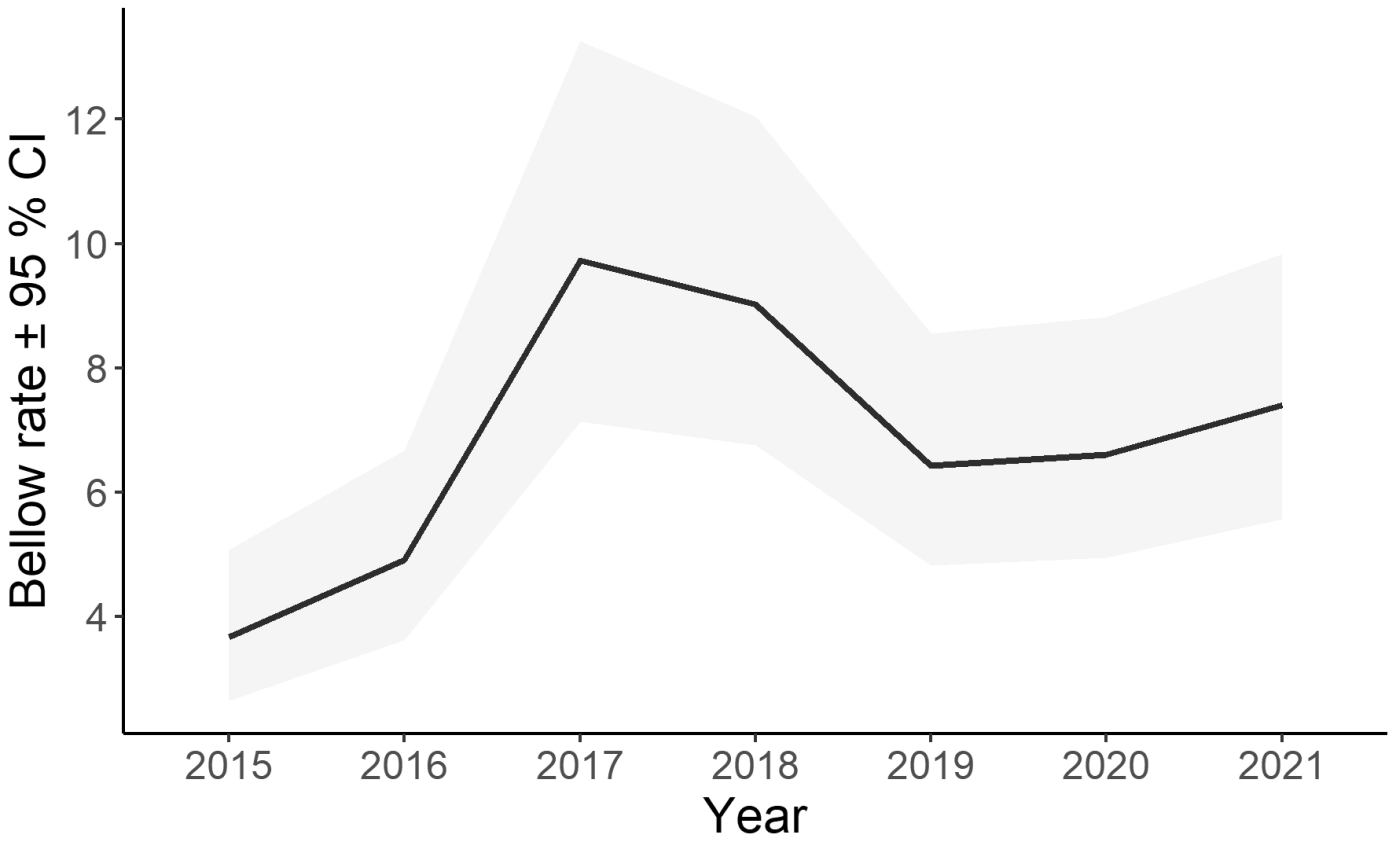
Line graph illustrating the annual trend for koala bellow rate (number of 10 min periods with a bellow per night).

A single model best explained variation in nightly bellow rate. Here, bellow rate varied with year of sampling (Table S6, Figure 10). Bellow rate was lowest in 2015 and doubled by 2017. A reduction in bellowing was observed in 2019 after fires and this level was maintained in 2020, with some recovery in 2021.

## 4 DISCUSSION

Using an extensive dataset comprising 224 sites spread across the 8.5 Mha study region we show that koala occupancy was stable over 7 years since 2015. This iconic species is typically cryptic, but acoustic sampling over many thousands of hours in our study combined with semi‐automated call recognition has proved exceptionally effective at detecting the species for systematic tracking of trends with a high level of precision. Occupancy was high (median ~0.75) over an extensive area of habitat and is indicative of a large meta‐population (Law et al., 2018). The stable trend was maintained despite a severe drought that led to megafires burning about 30% of their habitat in 2019 (Law, Gonsalves, Burgar, Brassil, Kerr, & O'Loughlin, 2022). Although occupancy was stable, bellow rate varied among years. As predicted based on other studies, regulated timber harvesting in state forests had no effect on the trend of either metric nor did land tenure (state forest vs. national park). The koala meta‐population in these public forests retains a high level of connectivity and genetic diversity (Johnson et al., 2018), which likely promotes resilience to these recent pressures at a regional scale.

There are a number of considerations when interpreting trends in koala occupancy. Acoustic monitoring is focused on male koalas, although several studies suggest that a 1:1 sex ratio is common in the species (L. Gonsalves, M. Blyton, B. Law, K. Brice, B. Moore and F. Wedrowicz, unpubl. data; Martin, 1985; Penn et al., 2000; Radford Miller, 2012; Watchorn & Whisson, 2019). We identified a stable trend in occupancy; however, acoustic arrays identified a reduction in koala density at sites burnt by moderate to high‐severity fire, even though those sites remained occupied (e.g. Law, Gonsalves, Burgar, Brassil, Kerr, & O'Loughlin, 2022). This means koala numbers can change before it is detected by occupancy. However, koalas typically occur at low density in NSW, and given SM4s sample a 300 m radius of forest (~28 ha) for koalas and male koala density in better quality forests of north‐east NSW is ~1 per 25 ha (Law, Gonsalves, Burgar, Brassil, Kerr, O'Loughlin, et al., 2022), then just a single male would be expected in the acoustic sample area where a bellow was recorded. Nonetheless, bellow rate is potentially a more sensitive metric than occupancy, and it varied over years. In our study, neither rainfall nor mean maximum temperature in the previous year influenced bellow rate. Although disturbance covariates were also not supported, bellow rate declined in 2019 after the Black Summer fires, but not as low as that recorded in 2015. It remains unclear why bellow rate was so low in 2015 and 2016 when we accounted for low detection probability in those years. Such large changes between consecutive years are unlikely to be due to changes in koala abundance in the absence of a major disturbance, suggesting changes in bellowing behaviour may be more likely, although the trigger remains unknown at this point. More research is needed to better understand the relationship between bellow rate and abundance as it is correlated at some sites (Hagens et al., 2018), but the relationship is variable elsewhere (Law et al., 2021).

### 4.1 Bellow detection probability per night

Although acoustic detection of koalas was highly successful, both sensor type and recogniser version influenced detection probability per night. AudioMoths had the poorest detection probability because their microphones are less sensitive than SM4s and SM‐minis (Hagens et al., 2018; Law, Kerr, et al., 2022). Detection probability for AudioMoths is much lower than we have recorded elsewhere (Law, Kerr, et al., 2022), possibly because of the low sample size here. Such variation in sensors is well known (Yip et al., 2017) but was accounted for when modelling occupancy. Different versions of a koala recogniser had a minor effect on detection probability, but the latest versions had better precision (fewer false positives, L. Gonsalves and B. Law, unpubl. data) and may have detected more bellows overall. These effects are accounted for when modelling occupancy, which is important given technology will continue to improve in the future. Previously minimum nightly temperature and rainfall were found to be negatively related to detection probability (Law et al., 2018, 2019), but these had negligible effects across this dataset relative to sensor type and recogniser version. Detection of any koala bellow (rather than on a nightly basis) was related to year of survey which incorporates variation in multiple factors, including sensor type, recogniser version and prevailing weather.

### 4.2 Initial occupancy and bellow rate

Dynamic occupancy modelling relies to a large extent on the strength of models for initial occupancy, because extinction and colonisation probabilities are applied to this base. The basis for our initial occupancy was derived by pooling sites across the first 3 years of our study, because these sites were not revisited until after that period. Although the climate naturally varied among these years, no extremes were experienced at that time, indicating a period of stability. Furthermore, a separate assessment of occupancy for the first 3 years of the study revealed no influence of year of sampling on occupancy (see Law et al., 2018). Initial occupancy was found to be related to elevation (−ve), NDVI (+ve) and selective timber harvesting 16–30 years prior (+ve). This is consistent with a previous single‐season analysis of a subset of these data where elevation was the primary driver, followed by NDVI, browse tree cover and fire (slight −ve) (Law et al., 2018). The present analysis included additional sites that occurred in lower‐quality koala habitat that were initially excluded from the Law et al. (2018) analysis. Low to mid‐elevation (below ~800 m) is well known to support a more extensive koala population in the north‐east of NSW (Kavanagh et al., 1995), though koalas do occur in localised areas on the higher elevation tablelands where suitable browse trees occur (Law et al., 2018). Also, some koala browse trees have greater concentrations of plant secondary metabolites at higher, colder elevations which might in part explain lower occupancy there (Moore et al., 2004). None of these covariates was found to influence bellow rate.

The positive relationship of occupancy with NDVI (canopy greenness) can be explained on the basis that this metric is an indicator of site productivity and leaf nutrition with koalas and other arboreal folivores responding favourably to this and leaf nutritional status (Moore et al., 2004; Stalenberg et al., 2014; Wu et al., 2019; Youngentob et al., 2015). It is worth noting that the relationship with NDVI does not always hold. For example, the relationship was inverse for koalas on private land in the study region, perhaps because koalas often occurred in more open grassy woodland or with scattered paddock trees on private land, which have lower NDVI than forests (Law, Slade, et al., 2022). Model support for both elevation and NDVI could explain why the koala habitat model had little influence on initial occupancy given both variables are important contributors to that model (Law, Caccamo, et al., 2017). Gardiner et al. (2023) found that koalas occurred more commonly in primary than secondary forests, but we found no relationship with mapped old growth. Similarly, tenure had no influence on occupancy or bellow rate and this is supported by a recent study that found koala density was broadly similar between state forest and national park (Law, Gonsalves, Burgar, Brassil, Kerr, O'Loughlin, et al., 2022). This result is not dissimilar from an assessment in south‐east Queensland/New England Tablelands, which found protected areas only enhanced koala occurrence by 10%, and the percentage of secondary forest did not modify the effect size (Terraube et al., 2023). Comparisons by tenure are coarse at best given timber harvesting in state forests now averages <50% of the tenure's landscape (Slade & Law, 2018) and it highlights that assessing the actual extent of harvesting in different age classes is more informative.

A minor positive relationship of occupancy with the extent of selective harvesting 16–30 years previously points to koala use of regrowth forest with medium‐sized trees embedded in a mosaic of harvest exclusions. Koalas have been associated previously with regrowth forest (Kavanagh et al., 1995), especially if uneven age (Radford Miller, 2012; Smith, 2004), which is a typical outcome of selective harvesting. In addition, radio‐tracking and scat searches have confirmed medium‐sized trees are the preferred‐size class used by koalas in the study area (Law, Slade, et al., 2022; McAlpine et al., 2023), perhaps because they are closely spaced, and the canopy is not as high as old growth, also noting tree hollows are not used by koalas. Other factors such as tenure, topography, site precipitation and temperature (though the latter is negatively correlated with elevation), soil phosphorus and moisture as well as disturbance variables (timber harvesting) had no detectable effect on initial occupancy, confirming koalas occur across a broad range of environments in the region (see also Goldingay et al., 2022). Other studies suggest soil phosphorus, water availability and/or tree species influence habitat use at a more local scale (Gardiner et al., 2023; McAlpine et al., 2023).

### 4.3 Extinction and colonisation probability

Extent of high‐severity fire was the only covariate to influence extinction probability and was the second top model that explained variation in colonisation probability. A similar, though steeper, pattern of extinction with high‐severity fire was found for yellow‐bellied gliders (*Petaurus australis*) in southern forests of NSW (Bilney et al., 2022). In NSW forests, the 2019/20 megafires were driven by extreme climate conditions rather than disturbance history (Bowman et al., 2021). Koalas were detected in 81% of burnt sites, although not in three where high‐severity fire dominated more than 50% of the site buffer (Bellangry and Kiwarrak State Forests and Talawahl Nature Reserve). One of these three sites was recolonised within 2 years of the fire (2021). At other burnt sites where koalas were detected, refuge areas occurred in the surrounding landscape, in that high fire severity covered <50% of the surrounding landscape and areas of unburnt forest were present or fire severity was lower. Koala density, as opposed to occupancy, is reduced substantially by high‐severity fire, with no koalas detected immediately after high‐severity fire (Law, Gonsalves, Burgar, Brassil, Kerr, & O'Loughlin, 2022). In contrast, low‐severity fire had no detectable effect on koala density, nor did pyrodiversity (Law, Gonsalves, Burgar, Brassil, Kerr, & O'Loughlin, 2022). More frequent fires were also identified in a maxent model as having a negative influence on koala habitat suitability (Law, Caccamo, et al., 2017). Variation in colonisation probability was best explained by annual rainfall. However, 14 models had some level of support, including the null that held colonisation probability constant. This is likely a reflection of the fact that occupancy from year‐to‐year was high with few sites available to become occupied from 1 year to the next. The extent of low‐severity fire had no influence (for extinction) or a lesser influence (for colonisation, as indicated by a model weight that was less than half of the top model).

The effect of severe fire contrasts with the effects of timber harvesting, which had no influence on extinction probability. This is likely because forestry operations are dispersed in time and space with <1% of 500 m buffers harvested in any given year compared to the Black Summer fires that burnt 36% of buffers. Importantly, environmental protections have increased over time from about 40% in the 20 years prior to 2018 (Slade & Law, 2018) to 50%–60% of the state forest landscape that is currently excluded from timber harvesting. Koala (male) density is a more sensitive metric than occupancy, but a before‐after harvesting experiment also found that density did not change after regulated harvesting, while density 5–10 years after heavy harvesting was also similar to control sites (Law, Gonsalves, Burgar, Brassil, Kerr, O'Loughlin, et al., 2022). Recent radio‐tracking has confirmed that male and female koalas make extensive use of young regenerating forest 5–10 years after harvesting (Law, Gonsalves, Burgar, Brassil, Kerr, O'Loughlin, et al., 2022). Koalas also experience high survival and no loss of condition after plantation harvesting when spotters are deployed so as to avoid harvesting where koalas are present (Hynes et al., 2021).

Years with high temperature or NDVI also had no detectable effect on extinction or colonisation (see also Goldingay et al., 2022) and indeed radio‐collared koalas continued to breed during the 2019 drought, despite severe, short‐term dieback in these forests (Law, Slade, et al., 2022). Resilience in the north‐east hinterland forests compared to other areas such as the hotter and drier Pilliga forests (Lunney et al., 2017), suggests our study region provides a more benign climate for koalas, noting the 2019 drought was intense but short‐lived (<2 years). Nonetheless, we suggest that climate change and the predicted increase in the frequency of high‐severity fire (Bowman et al., 2017; Clarke & Evans, 2019) will be the major threat to koalas in these forests. Our results also point to a potential negative effect on colonisation probability during years of extremely high rainfall and flooding.

### 4.4 Regional trend in occupancy

That koala occupancy was stable in these hinterland forests contrasts with expert opinion about declining koala populations in the broader region (estimated −50% change with 40% uncertainty; Adams‐Hosking et al., 2016). Yet our empirical results are consistent with mostly stable trends in two other local studies: one in Richmond Range National Park that did not vary with drought (2014–2021; Goldingay et al., 2022) and a second around Coffs Harbour as assessed by questionnaire surveys (1990–2011; Lunney et al., 2016). Together, these results emphasise that caution is needed when interpreting expert opinion, especially for cryptic species at a regional level, in comparison to the importance of systematic, empirically derived trends. A key difference between our stable trend and the expert‐derived −50% trend in this region is that our study focused only on public forests and only on the hinterland area and not the coastal strip. Although some of this public forest is harvested for timber under strict regulations, deforestation, a major threat for koalas, is not permitted unlike other areas in the region where urban development, roads, and dogs also have impacts (Lunney et al., 2002, 2017; McAlpine et al., 2015).

A stable trend in occupancy since 2015 also begs the question, ‘is stability a recent phenomenon?’ A lack of systematic data prevents a comprehensive answer to this, although a less precise, but nonetheless stable trend in occupancy was found using local stag watches in rural northern NSW between 1997 and 2014 (Law, Chidel, et al., 2017). Baseline occupancy estimates for fauna in the north‐east forests of NSW in the 1990s found that koala occupancy was 0.27 ± 0.17 based on 3000 spotlighting and call‐playback sites, the primary survey method used at the time (Kavanagh et al., 2022). While this suggests koala occupancy may have actually increased in this region since the 1990s, a very low detection probability of 0.09 per survey night and resulting poor precision suggests caution in interpreting these data. In addition, our sites focused on moderate to high‐quality koala habitat, with a smaller number of low‐quality sites. Thus, median estimates of occupancy derived from our sites are likely, on average, to represent better quality koala habitat than the extensive suite surveyed in the 1990s. There are no comparable data available prior to the 1990s, although Lunney et al. (2015) used historical records to argue that koalas were never abundant in the forests near Coffs Harbour.

In conclusion, our results highlight the recent stable trend in koala occupancy in north‐east NSW, but with variation among years in bellow rate including a decline after the 2019 Black Summer fires. They also emphasise the importance of systematic monitoring to derive rigorous trends and confirm the efficiency of passive acoustics for this purpose. Biodiversity monitoring will be increasingly important with a future of uncertain climate where species responses are not always predictable, especially in the face of climate‐driven changes to forest fire regimes. Ideally, given the scale of such an exercise, collaboration between key government and non‐government stakeholders would be best placed to deliver this important commitment to koala conservation.

## AUTHOR CONTRIBUTIONS

**Bradley Law:** Conceptualization (lead); funding acquisition (lead); investigation (equal); methodology (lead); project administration (lead); resources (lead); software (supporting); supervision (lead); validation (equal); visualization (equal); writing – original draft (lead); writing – review and editing (equal). **Traecey Brassil:** Conceptualization (supporting); data curation (lead); formal analysis (supporting); funding acquisition (supporting); investigation (equal); methodology (supporting); project administration (supporting); resources (supporting); software (supporting); supervision (supporting); validation (equal); visualization (supporting); writing – original draft (supporting); writing – review and editing (supporting). **Leroy Gonsalves:** Conceptualization (supporting); data curation (supporting); formal analysis (lead); funding acquisition (supporting); investigation (equal); methodology (supporting); project administration (supporting); software (supporting); supervision (supporting); validation (equal); visualization (equal); writing – original draft (supporting); writing – review and editing (equal). **Isobel Kerr:** Conceptualization (supporting); data curation (supporting); formal analysis (supporting); funding acquisition (supporting); investigation (equal); methodology (supporting); project administration (supporting); resources (supporting); software (supporting); supervision (supporting); validation (equal); visualization (supporting); writing – original draft (supporting); writing – review and editing (equal).

## CONFLICT OF INTEREST STATEMENT

The authors declare no conflicts of interest.

## Supporting information


Tables S1–S6

## Data Availability

Monitoring data and environmental variables used to generate the models are available on Dryad (https://datadryad.org/stash/share/3C_mTdEklu7a107aeaW5qfI1oP4fG5HMx3f5XrYaN5A).
