# Supplementary material for: Broad‐scale acoustic monitoring of koala populations suggests metapopulation stability, but varying bellow rate, in the face of major disturbances and climate extremes
Source: Ecol Evol. 2024 May 6;14(5):e11351. doi: 10.1002/ece3.11351 (PMC11074521; doi:10.1002/ece3.11351)
Supplement: Supplementary file 1 — Tables S1–S6 [file ECE3-14-e11351-s001.docx]

**Broad-scale acoustic monitoring of koala populations suggests metapopulation stability in the face of major disturbances and climate extremes**

Brad Law, Leroy Gonsalves, Traecey Brassil and Isobel Kerr

Forest Science, NSW DPI

**Full model section results**

Table S1. Model summary for detection probability.

| Model | daic | weight | npar | neg2ll |
| --- | --- | --- | --- | --- |
| psi(.),gam(.),eps(.),p(sensor) | 0 | 0.563 | 7 | 4689.61 |
| psi(.),gam(.),eps(.),p(sensor+recogniser) | 0.95 | 0.35 | 10 | 4684.56 |
| psi(.),gam(.),eps(.),p(sensor+month) | 3.76 | 0.086 | 9 | 4689.37 |
| psi(.),gam(.),eps(.),p(SEASON) | 19.67 | 0 | 8 | 4707.28 |
| psi(.),gam(.),eps(.),p(.) | 28.94 | 0 | 4 | 4724.55 |
| psi(.),gam(.),eps(.),p(max temp) | 29.13 | 0 | 5 | 4722.74 |
| psi(.),gam(.),eps(.),p(rain) | 29.13 | 0 | 5 | 4722.74 |
| psi(.),gam(.),eps(.),p(month) | 29.4 | 0 | 5 | 4723.01 |
| psi(.),gam(.),eps(.),p(recogniser) | 32.75 | 0 | 6 | 4724.36 |

Table S2. Model summary for Initial occupancy. Shaded rows indicate model support.

|  | Model | daic | weight | npar | neg2ll |
| --- | --- | --- | --- | --- | --- |
| Single-covariate | psi(DEM1000),gam(.),eps(.),p(sensor) | 0 | 0.4361 | 8 | 4670.38 |
|  | psi(DEM500),gam(.),eps(.),p(sensor) | 0.044 | 0.4266 | 8 | 4670.42 |
|  | psi(NDVI500),gam(.),eps(.),p(sensor) | 2.88 | 0.1033 | 8 | 4673.26 |
|  | psi(NDVI1000),gam(.),eps(.),p(sensor) | 6.1 | 0.0207 | 8 | 4676.48 |
|  | psi(mod1000),gam(.),eps(.),p(sensor) | 8.475 | 0.0063 | 8 | 4678.85 |
|  | psi(mod500),gam(.),eps(.),p(sensor) | 8.899 | 0.0051 | 8 | 4679.28 |
|  | psi(MLOG515500m),gam(.),eps(.),p(sensor) | 15.534 | 0.0002 | 8 | 4685.91 |
|  | psi(MLOG5500m),gam(.),eps(.),p(sensor) | 15.738 | 0.0002 | 8 | 4686.11 |
|  | psi(maxtmp),gam(.),eps(.),p(sensor) | 15.944 | 0.0002 | 8 | 4686.32 |
|  | psi(MLOG5151km),gam(.),eps(.),p(sensor) | 16.072 | 0.0001 | 8 | 4686.45 |
|  | psi(MLOG51km),gam(.),eps(.),p(sensor) | 16.384 | 0.0001 | 8 | 4686.76 |
|  | psi(SMIP),gam(.),eps(.),p(sensor) | 16.532 | 0.0001 | 8 | 4686.91 |
|  | psi(HLOG515500m),gam(.),eps(.),p(sensor) | 16.534 | 0.0001 | 8 | 4686.91 |
|  | psi(HLOG51km),gam(.),eps(.),p(sensor) | 16.536 | 0.0001 | 8 | 4686.91 |
|  | psi(MLOG1630500m),gam(.),eps(.),p(sensor) | 16.537 | 0.0001 | 8 | 4686.91 |
|  | psi(HLOG1630500m),gam(.),eps(.),p(sensor) | 16.541 | 0.0001 | 8 | 4686.92 |
|  | psi(.),gam(.),eps(.),p(sensor) | 17.234 | 0.0001 | 7 | 4689.61 |
|  | psi(tpi500),gam(.),eps(.),p(sensor) | 17.301 | 0.0001 | 8 | 4687.68 |
|  | psi(MLOG16301km),gam(.),eps(.),p(sensor) | 17.802 | 0.0001 | 8 | 4688.18 |
|  | psi(HCVOG1000),gam(.),eps(.),p(sensor) | 17.821 | 0.0001 | 8 | 4688.2 |
|  | psi(HCVOG500),gam(.),eps(.),p(sensor) | 18.068 | 0.0001 | 8 | 4688.45 |
|  | psi(tpi1000),gam(.),eps(.),p(sensor) | 18.612 | 0 | 8 | 4688.99 |
|  | psi(rough1000_1000),gam(.),eps(.),p(sensor) | 18.693 | 0 | 8 | 4689.07 |
|  | psi(rough100_500),gam(.),eps(.),p(sensor) | 18.808 | 0 | 8 | 4689.18 |
|  | psi(rough1000_500),gam(.),eps(.),p(sensor) | 18.938 | 0 | 8 | 4689.31 |
|  | psi(rough100_1000),gam(.),eps(.),p(sensor) | 19.092 | 0 | 8 | 4689.47 |
|  | psi(RF500),gam(.),eps(.),p(sensor) | 19.157 | 0 | 8 | 4689.53 |
|  | psi(pto500),gam(.),eps(.),p(sensor) | 19.195 | 0 | 8 | 4689.57 |
|  | psi(pto1000),gam(.),eps(.),p(sensor) | 19.224 | 0 | 8 | 4689.6 |
|  | psi(RF1000),gam(.),eps(.),p(sensor) | 19.231 | 0 | 8 | 4689.61 |
|  | psi(tenure),gam(.),eps(.),p(sensor) | 19.528 | 0 | 9 | 4687.91 |
|  | psi(rain),gam(.),eps(.),p(sensor) | 21.421 | 0 | 8 | 4691.8 |
|  | psi(HLOG16301km),gam(.),eps(.),p(sensor) | 21.764 | 0 | 8 | 4692.14 |
|  | psi(HLOG5500m),gam(.),eps(.),p(sensor) | 24.585 | 0 | 8 | 4694.96 |
|  | psi(HLOG5151km),gam(.),eps(.),p(sensor) | 24.894 | 0 | 8 | 4695.27 |
| Two-covariate additive | psi(DEM1000+NDVI500),gam(.),eps(.),p(sensor) | 0 | 0.534 | 9 | 4661.05 |
|  | psi(DEM1000+NDVI1000),gam(.),eps(.),p(sensor) | 2.43 | 0.1586 | 9 | 4663.47 |
|  | psi(DEM1000+mod500),gam(.),eps(.),p(sensor) | 5.74 | 0.0303 | 9 | 4666.78 |
|  | psi(DEM1000+mod1000),gam(.),eps(.),p(sensor) | 6.51 | 0.0206 | 9 | 4667.55 |
|  | psi(DEM1000+rough100_500),gam(.),eps(.),p(sensor) | 6.67 | 0.019 | 9 | 4667.72 |
|  | psi(DEM1000+rough100_1000),gam(.),eps(.),p(sensor) | 6.73 | 0.0184 | 9 | 4667.78 |
|  | psi(DEM1000),gam(.),eps(.),p(sensor) | 7.33 | 0.0137 | 8 | 4670.38 |
|  | psi(DEM1000+pto1000),gam(.),eps(.),p(sensor) | 7.47 | 0.0127 | 9 | 4668.52 |
|  | psi(DEM1000+maxtmp),gam(.),eps(.),p(sensor) | 7.6 | 0.0119 | 9 | 4668.65 |
|  | psi(DEM1000+RF500),gam(.),eps(.),p(sensor) | 7.74 | 0.0111 | 9 | 4668.79 |
|  | psi(DEM1000+MLOG515500m),gam(.),eps(.),p(sensor) | 7.87 | 0.0104 | 9 | 4668.91 |
|  | psi(DEM1000+MLOG5151km),gam(.),eps(.),p(sensor) | 7.92 | 0.0102 | 9 | 4668.96 |
|  | psi(DEM1000+MLOG5500m),gam(.),eps(.),p(sensor) | 7.92 | 0.0102 | 9 | 4668.97 |
|  | psi(DEM1000+pto500),gam(.),eps(.),p(sensor) | 8.18 | 0.009 | 9 | 4669.22 |
|  | psi(DEM1000+RF1000),gam(.),eps(.),p(sensor) | 8.31 | 0.0084 | 9 | 4669.35 |
|  | psi(DEM1000+HCVOG500),gam(.),eps(.),p(sensor) | 8.36 | 0.0082 | 9 | 4669.41 |
|  | psi(DEM1000+MLOG1630500m),gam(.),eps(.),p(sensor) | 8.42 | 0.0079 | 9 | 4669.46 |
|  | psi(DEM1000+HLOG1630500m),gam(.),eps(.),p(sensor) | 8.42 | 0.0079 | 9 | 4669.46 |
|  | psi(DEM1000+SMIP),gam(.),eps(.),p(sensor) | 8.42 | 0.0079 | 9 | 4669.46 |
|  | psi(DEM1000+rain),gam(.),eps(.),p(sensor) | 8.42 | 0.0079 | 9 | 4669.46 |
|  | psi(DEM1000+HLOG16301km),gam(.),eps(.),p(sensor) | 8.42 | 0.0079 | 9 | 4669.46 |
|  | psi(DEM1000+rough1000_500),gam(.),eps(.),p(sensor) | 8.47 | 0.0077 | 9 | 4669.52 |
|  | psi(DEM1000+tpi1000),gam(.),eps(.),p(sensor) | 8.57 | 0.0074 | 9 | 4669.61 |
|  | psi(DEM1000+HLOG515500m),gam(.),eps(.),p(sensor) | 8.57 | 0.0073 | 9 | 4669.62 |
|  | psi(DEM1000+HLOG5151km),gam(.),eps(.),p(sensor) | 8.59 | 0.0073 | 9 | 4669.64 |
|  | psi(DEM1000+rough1000_1000),gam(.),eps(.),p(sensor) | 8.6 | 0.0072 | 9 | 4669.64 |
|  | psi(DEM1000+MLOG51km),gam(.),eps(.),p(sensor) | 8.64 | 0.0071 | 9 | 4669.69 |
|  | psi(DEM1000+HLOG51km),gam(.),eps(.),p(sensor) | 8.65 | 0.0071 | 9 | 4669.7 |
|  | psi(DEM1000+HCVOG1000),gam(.),eps(.),p(sensor) | 8.74 | 0.0067 | 9 | 4669.79 |
|  | psi(DEM1000+HLOG5500m),gam(.),eps(.),p(sensor) | 8.75 | 0.0067 | 9 | 4669.79 |
|  | psi(DEM1000+tpi500),gam(.),eps(.),p(sensor) | 9.23 | 0.0053 | 9 | 4670.27 |
|  | psi(DEM1000+tenure),gam(.),eps(.),p(sensor) | 10.23 | 0.0032 | 10 | 4669.28 |
|  | psi(DEM1000+MLOG16301km),gam(.),eps(.),p(sensor) | 13.86 | 0.0005 | 9 | 4674.91 |
| Three-covariate additive | psi(DEM1000+NDVI500+MLOG16301km),gam(.),eps(.),p(sensor) | 0 | 0.4058 | 10 | 4654.63 |
|  | psi(DEM1000+NDVI500),gam(.),eps(.),p(sensor) | 4.41 | 0.0447 | 9 | 4661.05 |
|  | psi(DEM1000+NDVI500+pto1000),gam(.),eps(.),p(sensor) | 4.44 | 0.0441 | 10 | 4659.07 |
|  | psi(DEM1000+NDVI500+RF1000),gam(.),eps(.),p(sensor) | 4.75 | 0.0377 | 10 | 4659.39 |
|  | psi(DEM1000+NDVI500+pto500),gam(.),eps(.),p(sensor) | 5.46 | 0.0264 | 10 | 4660.1 |
|  | psi(DEM1000+NDVI500+RF500),gam(.),eps(.),p(sensor) | 5.63 | 0.0243 | 10 | 4660.26 |
|  | psi(DEM1000+NDVI500+HCVOG500),gam(.),eps(.),p(sensor) | 5.7 | 0.0235 | 10 | 4660.34 |
|  | psi(DEM1000+NDVI500+HCVOG1000),gam(.),eps(.),p(sensor) | 5.76 | 0.0228 | 10 | 4660.39 |
|  | psi(DEM1000+NDVI500+tpi500),gam(.),eps(.),p(sensor) | 5.85 | 0.0217 | 10 | 4660.49 |
|  | psi(DEM1000+NDVI500+SMIP),gam(.),eps(.),p(sensor) | 5.92 | 0.021 | 10 | 4660.56 |
|  | psi(DEM1000+NDVI500+HLOG16301km),gam(.),eps(.),p(sensor) | 5.95 | 0.0207 | 10 | 4660.58 |
|  | psi(DEM1000+NDVI500+rough100_500),gam(.),eps(.),p(sensor) | 6.25 | 0.0178 | 10 | 4660.89 |
|  | psi(DEM1000+NDVI500+MLOG5500m),gam(.),eps(.),p(sensor) | 6.26 | 0.0178 | 10 | 4660.89 |
|  | psi(DEM1000+NDVI500+mod1000),gam(.),eps(.),p(sensor) | 6.26 | 0.0177 | 10 | 4660.9 |
|  | psi(DEM1000+NDVI500+rain),gam(.),eps(.),p(sensor) | 6.26 | 0.0177 | 10 | 4660.9 |
|  | psi(DEM1000+NDVI500+MLOG1630500m),gam(.),eps(.),p(sensor) | 6.28 | 0.0175 | 10 | 4660.92 |
|  | psi(DEM1000+NDVI500+MLOG515500m),gam(.),eps(.),p(sensor) | 6.29 | 0.0175 | 10 | 4660.92 |
|  | psi(DEM1000+NDVI500+rough100_1000),gam(.),eps(.),p(sensor) | 6.3 | 0.0173 | 10 | 4660.94 |
|  | psi(DEM1000+NDVI500+HLOG5500m),gam(.),eps(.),p(sensor) | 6.31 | 0.0173 | 10 | 4660.95 |
|  | psi(DEM1000+NDVI500+rough1000_1000),gam(.),eps(.),p(sensor) | 6.38 | 0.0167 | 10 | 4661.01 |
|  | psi(DEM1000+NDVI500+MLOG5151km),gam(.),eps(.),p(sensor) | 6.38 | 0.0167 | 10 | 4661.02 |
|  | psi(DEM1000+NDVI500+HLOG515500m),gam(.),eps(.),p(sensor) | 6.39 | 0.0166 | 10 | 4661.02 |
|  | psi(DEM1000+NDVI500+HLOG5151km),gam(.),eps(.),p(sensor) | 6.4 | 0.0165 | 10 | 4661.04 |
|  | psi(DEM1000+NDVI500+HLOG1630500m),gam(.),eps(.),p(sensor) | 6.41 | 0.0165 | 10 | 4661.04 |
|  | psi(DEM1000+NDVI500+HLOG51km),gam(.),eps(.),p(sensor) | 6.41 | 0.0165 | 10 | 4661.04 |
|  | psi(DEM1000+NDVI500+maxtmp),gam(.),eps(.),p(sensor) | 6.41 | 0.0165 | 10 | 4661.04 |
|  | psi(DEM1000+NDVI500+rough1000_500),gam(.),eps(.),p(sensor) | 6.41 | 0.0165 | 10 | 4661.04 |
|  | psi(DEM1000+NDVI500+tpi1000),gam(.),eps(.),p(sensor) | 6.41 | 0.0164 | 10 | 4661.05 |
|  | psi(DEM1000+NDVI500+MLOG51km),gam(.),eps(.),p(sensor) | 7.38 | 0.0101 | 10 | 4662.02 |
|  | psi(DEM1000+NDVI500+tenure),gam(.),eps(.),p(sensor) | 8.34 | 0.0063 | 11 | 4660.97 |
|  | psi(DEM1000+NDVI500+mod500),gam(.),eps(.),p(sensor) | 11.27 | 0.0014 | 10 | 4665.9 |

Table S3. Model summary for colonisation probability. Shaded rows indicate model support.

| Model | daic | weight | npar | neg2ll |
| --- | --- | --- | --- | --- |
| psi(DEM1000+NDVI500+MLOG16301km),eps(.),gam(rain),p(sensor) | 0 | 0.0858 | 11 | 4651.59 |
| psi(DEM1000+NDVI500+MLOG16301km),eps(.),gam(HVH500m),p(sensor) | 0.44 | 0.0687 | 11 | 4652.04 |
| psi(DEM1000+NDVI500+MLOG16301km),eps(.),gam(HLOGg30500m),p(sensor) | 0.6 | 0.0635 | 11 | 4652.2 |
| psi(DEM1000+NDVI500+MLOG16301km),eps(.),gam(HVH1km),p(sensor) | 0.83 | 0.0568 | 11 | 4652.42 |
| psi(DEM1000+NDVI500+MLOG16301km),eps(.),gam(MLOG515500m),p(sensor) | 0.91 | 0.0544 | 11 | 4652.51 |
| psi(DEM1000+NDVI500+MLOG16301km),eps(.),gam(HLOGg301km),p(sensor) | 1.01 | 0.0519 | 11 | 4652.6 |
| psi(DEM1000+NDVI500+MLOG16301km),eps(.),gam(.),p(sensor) | 1.04 | 0.051 | 10 | 4654.63 |
| psi(DEM1000+NDVI500+MLOG16301km),eps(.),gam(MLOG5151km),p(sensor) | 1.2 | 0.047 | 11 | 4652.8 |
| psi(DEM1000+NDVI500+MLOG16301km),eps(.),gam(MLOG1630500m),p(sensor) | 1.38 | 0.043 | 11 | 4652.98 |
| psi(DEM1000+NDVI500+MLOG16301km),eps(.),gam(HLOG515500m),p(sensor) | 1.54 | 0.0397 | 11 | 4653.14 |
| psi(DEM1000+NDVI500+MLOG16301km),eps(.),gam(HLOG5151km),p(sensor) | 1.54 | 0.0396 | 11 | 4653.14 |
| psi(DEM1000+NDVI500+MLOG16301km),eps(.),gam(MLOG5500m),p(sensor) | 1.7 | 0.0366 | 11 | 4653.3 |
| psi(DEM1000+NDVI500+MLOG16301km),eps(.),gam(MLOGg30500m),p(sensor) | 1.76 | 0.0355 | 11 | 4653.36 |
| psi(DEM1000+NDVI500+MLOG16301km),eps(.),gam(MLOG51km),p(sensor) | 1.82 | 0.0345 | 11 | 4653.42 |
| psi(DEM1000+NDVI500+MLOG16301km),eps(.),gam(MLOG16301km),p(sensor) | 1.88 | 0.0336 | 11 | 4653.47 |
| psi(DEM1000+NDVI500+MLOG16301km),eps(.),gam(LOW1km),p(sensor) | 2.01 | 0.0313 | 11 | 4653.61 |
| psi(DEM1000+NDVI500+MLOG16301km),eps(.),gam(NDVI500),p(sensor) | 2.02 | 0.0312 | 11 | 4653.62 |
| psi(DEM1000+NDVI500+MLOG16301km),eps(.),gam(HLOG1630500m),p(sensor) | 2.14 | 0.0294 | 11 | 4653.74 |
| psi(DEM1000+NDVI500+MLOG16301km),eps(.),gam(HLOG51km),p(sensor) | 2.14 | 0.0294 | 11 | 4653.74 |
| psi(DEM1000+NDVI500+MLOG16301km),eps(.),gam(LOW500m),p(sensor) | 2.14 | 0.0294 | 11 | 4653.74 |
| psi(DEM1000+NDVI500+MLOG16301km),eps(.),gam(HLOG5500m),p(sensor) | 2.15 | 0.0293 | 11 | 4653.74 |
| psi(DEM1000+NDVI500+MLOG16301km),eps(.),gam(HLOG16301km),p(sensor) | 2.83 | 0.0209 | 11 | 4654.42 |
| psi(DEM1000+NDVI500+MLOG16301km),eps(.),gam(SMIP),p(sensor) | 2.94 | 0.0197 | 11 | 4654.54 |
| psi(DEM1000+NDVI500+MLOG16301km),eps(.),gam(MLOGg301km),p(sensor) | 3.06 | 0.0186 | 11 | 4654.65 |
| psi(DEM1000+NDVI500+MLOG16301km),eps(.),gam(NDVI1000),p(sensor) | 3.06 | 0.0186 | 11 | 4654.66 |
| psi(DEM1000+NDVI500+MLOG16301km),eps(.),gam(SEASON_corrected),p(sensor) | 11.23 | 0.0003 | 12 | 4660.83 |
| psi(DEM1000+NDVI500+MLOG16301km),eps(.),gam(maxtemp),p(sensor) | 15.53 | 0 | 11 | 4667.12 |

Table S4. Model summary for extinction probability. Shaded rows indicate model support.

| Model | daic | weight | npar | neg2ll |
| --- | --- | --- | --- | --- |
| psi(DEM1000+NDVI500+MLOG16301km),gam(.),eps(HVH500m),p(sensor) | 0 | 0.5213 | 11 | 4646.45 |
| psi(DEM1000+NDVI500+MLOG16301km),gam(.),eps(HVH1km),p(sensor) | 1.11 | 0.2998 | 11 | 4647.56 |
| psi(DEM1000+NDVI500+MLOG16301km),gam(.),eps(MLOGg30500m),p(sensor) | 7.15 | 0.0146 | 11 | 4653.6 |
| psi(DEM1000+NDVI500+MLOG16301km),gam(.),eps(SMIP),p(sensor) | 7.44 | 0.0127 | 11 | 4653.89 |
| psi(DEM1000+NDVI500+MLOG16301km),gam(.),eps(rainfall),p(sensor) | 7.46 | 0.0125 | 11 | 4653.92 |
| psi(DEM1000+NDVI500+MLOG16301km),gam(.),eps(MLOG1630500m),p(sensor) | 7.59 | 0.0117 | 11 | 4654.04 |
| psi(DEM1000+NDVI500+MLOG16301km),gam(.),eps(HLOG1630500m),p(sensor) | 7.59 | 0.0117 | 11 | 4654.04 |
| psi(DEM1000+NDVI500+MLOG16301km),gam(.),eps(MLOG515500m),p(sensor) | 7.59 | 0.0117 | 11 | 4654.04 |
| psi(DEM1000+NDVI500+MLOG16301km),gam(.),eps(HLOG16301km),p(sensor) | 8.03 | 0.0094 | 11 | 4654.49 |
| psi(DEM1000+NDVI500+MLOG16301km),gam(.),eps(maxtemp),p(sensor) | 8.16 | 0.0088 | 11 | 4654.62 |
| psi(DEM1000+NDVI500+MLOG16301km),gam(.),eps(MLOGg301km),p(sensor) | 8.22 | 0.0085 | 11 | 4654.68 |
| psi(DEM1000+NDVI500+MLOG16301km),gam(.),eps(MLOG5500m),p(sensor) | 8.85 | 0.0063 | 11 | 4655.3 |
| psi(DEM1000+NDVI500+MLOG16301km),gam(.),eps(MLOG51km),p(sensor) | 8.88 | 0.0061 | 11 | 4655.34 |
| psi(DEM1000+NDVI500+MLOG16301km),gam(.),eps(HLOGg30500m),p(sensor) | 8.91 | 0.0061 | 11 | 4655.36 |
| psi(DEM1000+NDVI500+MLOG16301km),gam(.),eps(HLOG5500m),p(sensor) | 9.09 | 0.0055 | 11 | 4655.55 |
| psi(DEM1000+NDVI500+MLOG16301km),gam(.),eps(LOW1km),p(sensor) | 9.17 | 0.0053 | 11 | 4655.63 |
| psi(DEM1000+NDVI500+MLOG16301km),gam(.),eps(LOW500m),p(sensor) | 9.19 | 0.0053 | 11 | 4655.65 |
| psi(DEM1000+NDVI500+MLOG16301km),gam(.),eps(HLOG51km),p(sensor) | 9.2 | 0.0053 | 11 | 4655.65 |
| psi(DEM1000+NDVI500+MLOG16301km),gam(.),eps(NDVI500),p(sensor) | 9.35 | 0.0049 | 11 | 4655.8 |
| psi(DEM1000+NDVI500+MLOG16301km),gam(.),eps(MLOG5151km),p(sensor) | 9.39 | 0.0048 | 11 | 4655.85 |
| psi(DEM1000+NDVI500+MLOG16301km),gam(.),eps(HLOG5151km),p(sensor) | 9.79 | 0.0039 | 11 | 4656.24 |
| psi(DEM1000+NDVI500+MLOG16301km),gam(.),eps(MLOG16301km),p(sensor) | 9.81 | 0.0039 | 11 | 4656.26 |
| psi(DEM1000+NDVI500+MLOG16301km),gam(.),eps(NDVI1000),p(sensor) | 9.87 | 0.0037 | 11 | 4656.32 |
| psi(DEM1000+NDVI500+MLOG16301km),gam(.),eps(HLOG515500m),p(sensor) | 9.9 | 0.0037 | 11 | 4656.35 |
| psi(DEM1000+NDVI500+MLOG16301km),gam(.),eps(HLOGg301km),p(sensor) | 9.92 | 0.0037 | 11 | 4656.37 |
| psi(DEM1000+NDVI500),gam(.),eps(.),p(sensor) | 10.59 | 0.0026 | 9 | 4661.05 |
| psi(DEM1000+NDVI500+MLOG16301km),gam(.),eps(SEASON_corrected),p(sensor) | 17.98 | 0.0001 | 12 | 4662.43 |

Table S5. Model summary for N-mixture model detection probability. Shaded rows indicate model support.

| Model | elpd | nparam | elpd_diff | se_diff |
| --- | --- | --- | --- | --- |
| P~Year, N~1 + (1\|Site) | -11975.5 | 584.676 | 0 | 0 |
| P~Sensor, N~1 + (1\|Site) | -12053.7 | 579.673 | -78.138 | 110.939 |
| P~Recogniser, N~1 + (1\|Site) | -12135.6 | 587.294 | -160.113 | 73.933 |
| P~1, N~1 + (1\|Site) | -12231.8 | 563.917 | -256.257 | 90.746 |
| P~Month, N~1 + (1\|Site) | -12234.9 | 584.408 | -259.427 | 91.843 |

Table S6. Model summary for N-mixture model bellow rate. Shaded rows indicate model support.

| Model | elpd | nparam | elpd_diff | se_diff |
| --- | --- | --- | --- | --- |
| P~Year, N~Year + (1\|Site) | -11920.3 | 581.137 | 0 | 0 |
| P~Year, N~LOW500m + (1\|Site) | -11952.9 | 579.354 | -32.547 | 28.365 |
| P~Year, N~maxtmp + (1\|Site) | -11964.8 | 575.572 | -44.526 | 25.897 |
| P~Year, N~MLOG51km + (1\|Site) | -11966 | 577.253 | -45.729 | 25.937 |
| P~Year, N~LOW1km + (1\|Site) | -11966.5 | 588.902 | -46.231 | 27.015 |
| P~Year, N~tpi1000 + (1\|Site) | -11969.6 | 576.436 | -49.29 | 26.742 |
| P~Year, N~HLOGg30500m + (1\|Site) | -11970 | 578.495 | -49.728 | 26.452 |
| P~Year, N~MLOG5151km + (1\|Site) | -11970.3 | 580.776 | -49.981 | 26.166 |
| P~Year, N~pto1000 + (1\|Site) | -11970.7 | 579.215 | -50.401 | 27.423 |
| P~Year, N~MLOG1630500m + (1\|Site) | -11970.9 | 590.727 | -50.547 | 26.767 |
| P~Year, N~NDVI1000 + (1\|Site) | -11971.7 | 586.45 | -51.404 | 27.179 |
| P~Year, N~mod500 + (1\|Site) | -11971.7 | 583.398 | -51.419 | 26.979 |
| P~Year, N~rough1000_500 + (1\|Site) | -11971.8 | 582.368 | -51.451 | 26.822 |
| P~Year, N~HVH1km + (1\|Site) | -11971.9 | 588.302 | -51.541 | 28.013 |
| P~Year, N~NDVI500 + (1\|Site) | -11972.2 | 591.804 | -51.914 | 27.022 |
| P~Year, N~HLOG16301km + (1\|Site) | -11972.7 | 583.589 | -52.421 | 26.398 |
| P~Year, N~tpi500 + (1\|Site) | -11973.4 | 586.781 | -53.086 | 27.402 |
| P~Year, N~HLOGg301km + (1\|Site) | -11973.5 | 583.08 | -53.165 | 26.814 |
| P~Year, N~rough1000_1000 + (1\|Site) | -11973.5 | 577.729 | -53.222 | 26.573 |
| P~Year, N~RF1000 + (1\|Site) | -11974 | 580.787 | -53.644 | 27.459 |
| P~Year, N~rough100_1000 + (1\|Site) | -11974.1 | 582.962 | -53.742 | 26.792 |
| P~Year, N~HLOG515500m + (1\|Site) | -11975 | 582.889 | -54.694 | 27.183 |
| P~Year, N~MLOG515500m + (1\|Site) | -11975 | 581.415 | -54.709 | 27.062 |
| P~Year, N~HLOG5500m + (1\|Site) | -11975.4 | 588.821 | -55.051 | 27.415 |
| P~Year, N~1 + (1\|Site) | -11975.5 | 584.676 | -55.2 | 27.509 |
| P~Year, N~HLOG1630500m + (1\|Site) | -11975.6 | 583.335 | -55.295 | 26.646 |
| P~Year, N~HVH500m + (1\|Site) | -11975.9 | 589.414 | -55.553 | 28.83 |
| P~Year, N~HLOG51km + (1\|Site) | -11976.4 | 585.79 | -56.121 | 27.833 |
| P~Year, N~DEM500 + (1\|Site) | -11977.1 | 588.466 | -56.794 | 27.543 |
| P~Year, N~rain + (1\|Site) | -11978 | 591.173 | -57.71 | 26.866 |
| P~Year, N~rough100_500 + (1\|Site) | -11978.2 | 588.17 | -57.858 | 26.876 |
| P~Year, N~pto500 + (1\|Site) | -11978.3 | 587.743 | -57.982 | 27.324 |
| P~Year, N~DEM1000 + (1\|Site) | -11978.3 | 587.042 | -57.989 | 26.747 |
| P~Year, N~MLOGg30500m + (1\|Site) | -11978.6 | 589.541 | -58.301 | 27.07 |
| P~Year, N~MLOGg301km + (1\|Site) | -11979 | 595.951 | -58.688 | 26.847 |
| P~Year, N~RF500 + (1\|Site) | -11980.8 | 586.527 | -60.496 | 27.487 |
| P~Year, N~MLOG16301km + (1\|Site) | -11981.1 | 589.991 | -60.808 | 27.218 |
| P~Year, N~MLOG5500m + (1\|Site) | -11981.2 | 589.996 | -60.847 | 26.978 |
| P~Year, N~HCVOG1000 + (1\|Site) | -11983.4 | 594.467 | -63.118 | 27.417 |
| P~Year, N~HLOG5151km + (1\|Site) | -11983.5 | 592.237 | -63.168 | 27.597 |
| P~Year, N~mod1000 + (1\|Site) | -11985.2 | 598 | -64.844 | 28.147 |
| P~Year, N~HCVOG500 + (1\|Site) | -11990.9 | 605.796 | -70.604 | 27.515 |
